# Advanced biomedical materials and drug delivery systems for gastrointestinal applications: current understanding and future perspectives

**DOI:** 10.1186/s43556-026-00654-7

**Published:** 2026-11-13

**Authors:** Yijie Hu, Shengjie Luo, Zixin Chen, Rongkang Huang, Feng Wang, Yong Li, Jian Cai

**Affiliations:** 1 Department of Colorectal Surgery, Shenzhen Second People’s Hospital, First Affiliated Hospital of Shenzhen University, Medical Innovation Technology Transformation Center of Shenzhen Second People’s Hospital, Shenzhen University, Shenzhen, 518035 China; 2 Colorectal Surgery Unit III, Guangdong Institute of Gastroenterology, Guangzhou (Huangpu) Zhongliu Biomedical Innovation Center, Key Laboratory of Human Microbiome and Chronic Diseases, Sun Yat-sen University, Ministry of Education, Guangdong Provincial Key Laboratory of Colorectal and Pelvic Floor Diseases, The Sixth Affiliated Hospital, Sun Yat-sen University, Guangzhou, 510655 China; 3 Medical Research Institute, Guangdong Provincial People’s Hospital, Guangdong Academy of Medical Sciences, Southern Medical University, Guangzhou, 510080 China; 4 Department of Gastrointestinal Surgery, Department of General Surgery, Guangdong Provincial People’s Hospital, Guangdong Academy of Medical Sciences, Southern Medical University, Guangzhou, 510080 China; 5 Guangdong Provincial People’s Hospital Ganzhou Hospital, Ganzhou Municipal Hospital, Ganzhou, 341000 China

**Keywords:** Gastrointestinal barriers, Biomedical materials, Drug delivery, Clinical translation

## Abstract

The gastrointestinal (GI) tract is a major site of ulcerative, inflammatory, and neoplastic diseases. It also functions as an important interface in systemic metabolic regulation. The effectiveness of current treatments is often limited by poor drug stability and short residence times in the GI tract, insufficient exposure at the target site, and unnecessary systemic exposure. These limitations are compounded by regional pH changes, digestive enzymes, mucus turnover, peristaltic clearance, immune surveillance, and microbial metabolism. Advanced biomedical materials, including mucoadhesive hydrogels, stimuli-responsive nanoplatforms, metal–organic frameworks (MOFs), and extracellular matrix (ECM)-derived scaffolds, are increasingly being developed to protect cargo, prolong mucosal residence, enable site-selective release, support tissue repair, and modulate immune or microbial processes. However, selecting a suitable material and delivery strategy for different GI diseases remains challenging. This review examines physiological barriers to drug delivery in the GI tract, classifies advanced biomaterials and delivery systems by therapeutic function and platform type, and evaluates their applications across GI diseases with attention to administration routes, target sites, therapeutic outcomes, and clinical translation. Finally, we discuss future priorities for route-specific material design, human-relevant evaluation, and the safe development of advanced delivery systems. This review aims to provide a practical reference for selecting appropriate biomaterials and delivery systems for GI disease treatment and for guiding their further development toward clinical use.

## Introduction

Gastrointestinal (GI) diseases encompass common nonmalignant disorders, chronic inflammatory conditions, and major malignancies. Together, they impose a substantial global health burden. Based on the reported 2024 estimates for each cancer type, colorectal cancer (CRC) and stomach cancer together accounted for approximately 3.0 million new cases and 1.56 million deaths worldwide; CRC ranked third in incidence and second in mortality, whereas stomach cancer ranked fifth for both [1]. The epidemiology of inflammatory bowel disease (IBD) is also changing. An analysis of 522 population-based studies across 82 regions showed that prevalence continues to increase in many early-industrialized regions, while incidence is rising in many newly industrialized regions [2]. Peptic ulcer disease (PUD) also contributes to this burden, with reported annual prevalence estimates ranging from 0.12% to 1.5% [3]. Beyond these localized disorders, the GI tract contributes to systemic metabolic regulation through nutrient processing, endocrine signaling, barrier function, and interactions between the resident microbiota and the host [4–7]. This broad disease spectrum creates distinct therapeutic requirements, including protecting labile cargo, eradicating *Helicobacter pylori (H. pylori)*, controlling mucosal inflammation, sealing and repairing tissue defects, delivering antitumor agents, and modulating gut-associated metabolic pathways.

A primary challenge in developing biomaterials and drug delivery systems for GI diseases is the highly variable regional environment along the GI tract. Regional pH, digestive enzymes, mucus, peristaltic clearance, epithelial transport, and microbial metabolism can alter drug stability, residence, absorption, and target-site exposure [8, 9]. The relevance of each barrier depends on the administration route and target compartment. Oral systems may need to protect cargo during transit and release it at the intended site. Endoscopically delivered materials must be compatible with the delivery device, whereas materials intended for local retention may need to adhere to wet, moving tissue surfaces. Systemic carriers must remain in circulation long enough to reach the intended lesion. The material and delivery-platform classes considered in this review include hydrogels, lipid-based carriers, polymeric and inorganic nanoparticles, porous coordination materials such as metal–organic frameworks (MOFs), cell membrane-coated carriers, and extracellular matrix (ECM)-derived scaffolds. Across these platforms, composition and architecture can be adjusted to support cargo protection, mucosal residence or penetration, site-selective release, tissue repair, and immune or microbiome modulation. Hydrogels offer tunable mechanical properties and release behavior, whereas ECM-derived scaffolds provide structural and biochemical cues for tissue repair [10–12]. Other design features, including pH responsiveness and cell membrane coating, can be used to adjust adhesion or release and to modify immune recognition and tissue interactions according to the local environment and intended route of administration [13, 14]. For systemically administered tumor nanocarriers, distribution may also be influenced by the enhanced permeability and retention (EPR) effect, although its magnitude varies with tumor biology and carrier properties [15, 16]. Research on disease-specific nanocarriers, smart oral devices, and micro/nanomotors has further expanded the range of strategies available for GI delivery [8, 17–19].

**Fig. 1:**
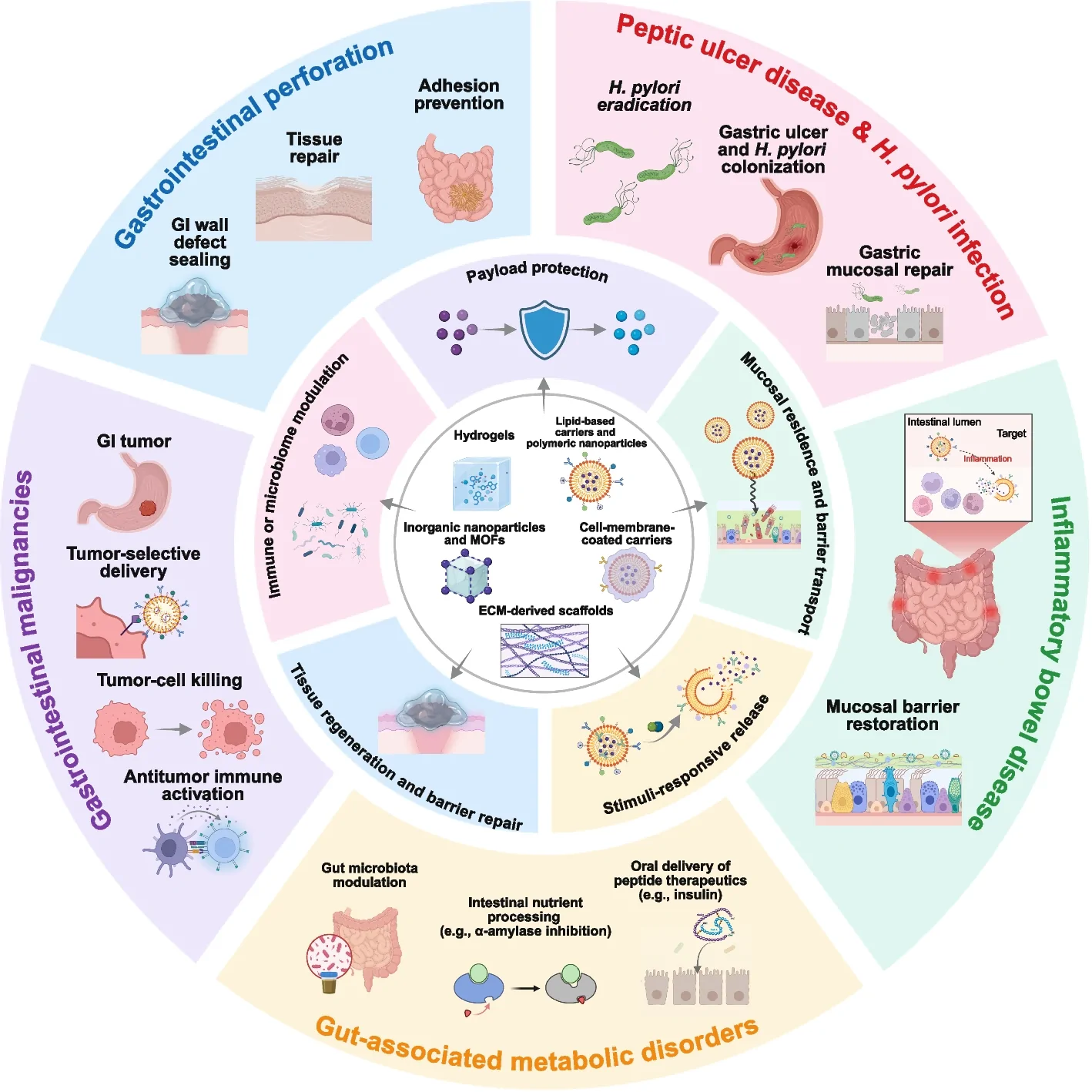
Representative gastrointestinal biomaterial platforms, functions, and disease-oriented applications. The center presents five representative classes of biomaterial platforms, the middle ring groups their five principal functions in gastrointestinal applications, and the outer ring shows how these functions are emphasized differently across gastrointestinal diseases and gut-associated metabolic disorders. Abbreviations: ECM, extracellular matrix; GI, gastrointestinal; H. pylori, Helicobacter pylori; MOFs, metal–organic frameworks. Created in BioRender

This review is organized around GI physiological barriers to drug delivery, the classification and design of biomaterials and delivery systems, disease-specific applications, and clinical translation. We first describe regional pH and enzymatic exposure, mucus dynamics and peristaltic clearance, epithelial and immune barriers, and microbiome complexity. We then classify biomaterials by their predominant therapeutic functions and compare the major material and delivery-platform classes. Representative biomaterial platforms, principal functions, and disease-oriented applications are summarized in Fig. 1. The disease-specific analysis covers PUD, *H. pylori* infection, GI perforation, IBD, GI malignancies, and gut-associated metabolic disorders, with attention to the study model, administration route, therapeutic outcomes, and limitations. The translational analysis covers animal-to-human differences, long-term material fate and biosafety, manufacturing and quality control, regulatory classification, and available clinical evidence. Finally, we discuss future priorities in artificial intelligence (AI)-assisted material design, human-relevant organ-on-chip testing, and controllable engineered living materials. By considering these dimensions together, this review aims to provide a practical basis for matching biomaterial design to the intended GI indication and administration route, while identifying the evidence still needed for clinical translation.

## Current understanding of GI physiological barriers for drug delivery

The GI tract contains chemical, physical, epithelial, immune, and microbial barriers that protect the host from ingested pathogens and toxins but can also limit drug stability, residence, transport, and absorption [8].

### pH gradients and enzymatic degradation

The GI tract shows marked regional variation in luminal pH. In healthy adults, gastric pH is strongly acidic under fasting conditions. Food buffering temporarily raises gastric pH, after which it returns toward fasting values as acid secretion continues [20]. Beyond the stomach, mean luminal pH increases to approximately 6.6 in the proximal small intestine and reaches about 7.5 in the terminal ileum. Upon entry into the cecum, pH decreases to approximately 6.4 and subsequently rises toward 7.0 in the distal colon [21].

Regional differences in pH and enzyme distribution create distinct chemical environments along the GI tract that can reduce the bioavailability of orally administered drugs. Regional pH can alter drug ionization, dissolution, stability, and carrier release. Pepsin acts in the acidic stomach, whereas pancreatic proteases such as trypsin and chymotrypsin, together with brush-border peptidases, act in the small intestine. Microbial enzymes can also transform susceptible drugs and materials in the colon [8, 22, 23]. Unprotected peptide and protein therapeutics, including insulin and antibody-based drugs, typically have oral bioavailability below 1% because of luminal degradation and poor epithelial permeability [24].

For oral delivery, a formulation must protect its cargo during gastric transit without preventing release in the intended intestinal compartment. Regional pH changes and digestive enzymes can destabilize both the carrier and its payload, whereas an overly persistent protective layer can delay release and reduce access to the target tissue. Effective formulations must therefore balance protection during transit with release at the intended site [8].

### Mucus barrier and peristaltic clearance

Most luminal surfaces of the stomach and intestines are covered by mucus, which separates luminal contents from the underlying epithelium [25]. Highly glycosylated mucins form a viscoelastic network that can hinder particulate carriers through steric obstruction and adhesive interactions [26]. The extent of this restriction depends on the anatomical site, particle size, and surface chemistry [27–30]. Mucus is continuously secreted and cleared, and intestinal motility can further shorten the mucosal residence time of oral formulations [8, 29].

Intact epithelial junctions restrict paracellular transport of macromolecules, while limited cellular uptake and intracellular trafficking can restrict transcellular delivery [22, 24]. For susceptible drug molecules, intestinal efflux transporters can further reduce absorption [8]. Endoscopically applied adhesives and sealants must pass through the instrument channel, conform to moving tissue, and retain wet-tissue adhesion during exposure to luminal fluid and peristaltic motion [8, 13].

### Immune surveillance and microbiome complexity

The GI tract is a major immune interface in the human body and contains intraepithelial lymphocytes, immune cells in the lamina propria, gut-associated lymphoid tissue, and secretory immunoglobulin A [31]. This immune network provides active surveillance against pathogens but can also recognize administered materials. For instance, scaffolds may activate local innate immune responses, whereas systemically administered nanoparticles may undergo phagocytic clearance [32–34].

The GI tract contains on the order of 10^13^ bacterial cells [35]. Microbial enzymes can cleave azo bonds and degrade certain polysaccharides and other fermentable materials, whereas microbial metabolites can influence local pH, redox conditions, and immune activity [4, 5, 8, 36]. These processes may alter the stability or release behavior of a susceptible carrier before it reaches the target site. The same microbial cues can also be used to trigger colon-specific drug release. Because microbiome composition and metabolic activity vary substantially among individuals, microbiota-responsive systems may show variable degradation and release profiles across patients [5, 8].

Materials face different biological challenges depending on whether they remain in the GI lumen or enter tissues and the systemic circulation. Carriers that enter damaged tissue or the systemic circulation are more likely to undergo innate immune recognition and macrophage clearance. By contrast, the performance and safety of nonabsorbed luminal systems depend mainly on local mucosal tolerability and compatibility with resident microbiota.

### Common mechanisms for overcoming GI barriers

Although the physiological barriers encountered vary across GI regions and routes of administration, the functions required of delivery systems can be grouped into five broad categories. These functions need not be incorporated into every system; instead, they should be selected and combined according to the administration route, cargo properties, and target site. First, payloads susceptible to gastric acid, enzymes, or bile salts require protection during GI transit [8]. Second, interactions with mucus or wet tissue can be adjusted to control distribution and residence at the target site [29, 37]. Third, regional pH, enzymatic activity, reactive oxygen species (ROS), or microbial metabolism can serve as release triggers under selected GI or disease-associated conditions [8, 38]. Fourth, delivery to the intended tissue or cells may require epithelial transport, lesion binding, or cellular uptake [33, 39]. Fifth, the material should have a predictable fate after treatment through degradation, elimination, or planned removal, as appropriate [40].

**Fig. 2:**
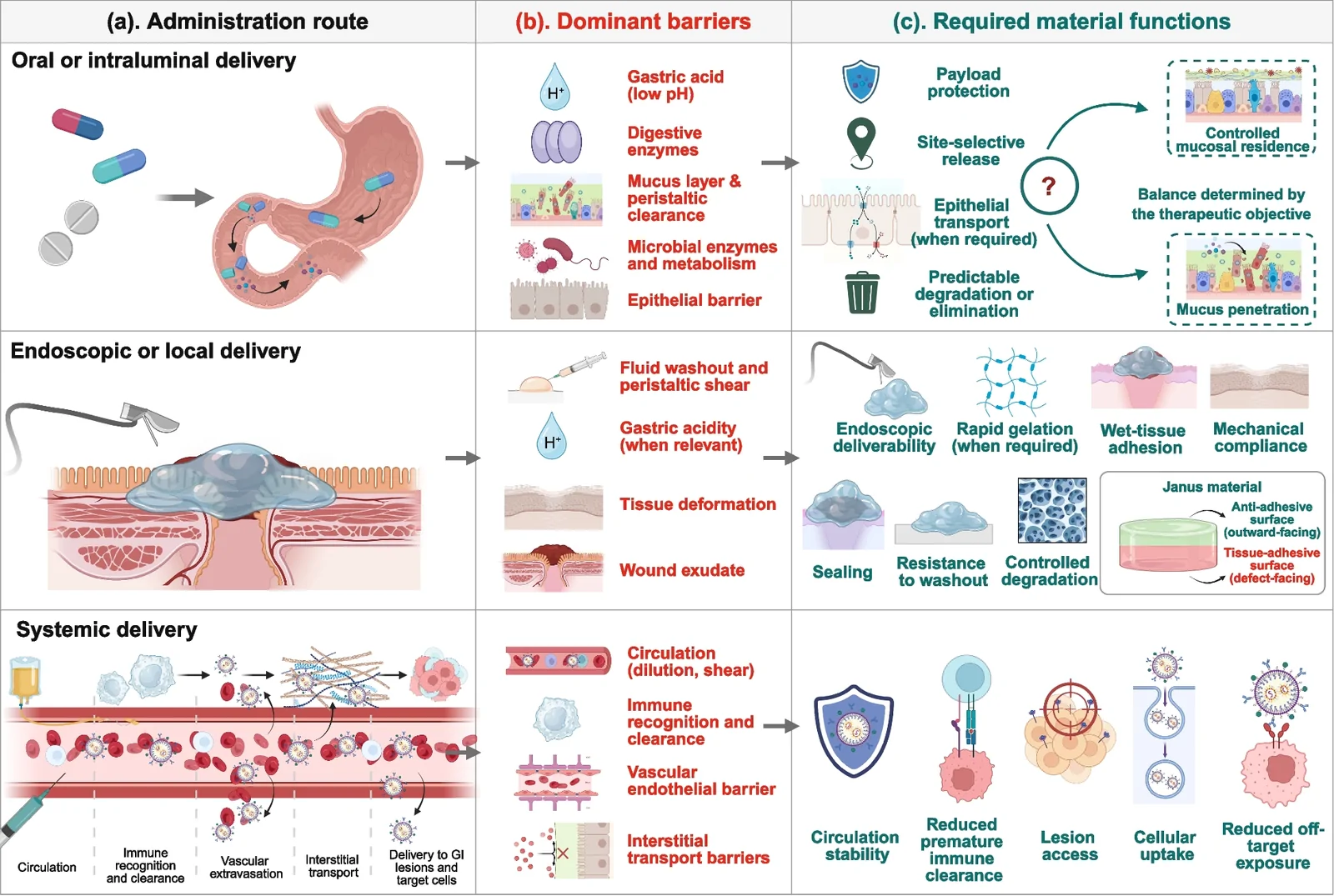
Route-specific barriers and material requirements for gastrointestinal delivery. **a** The principal administration routes considered are oral or intraluminal, endoscopic or local, and systemic delivery. **b** Each route presents a distinct set of physiological and delivery barriers. **c** The required material functions are tailored to the barriers associated with each route. Abbreviation: GI, gastrointestinal. Created in BioRender

Material requirements differ across administration routes. Oral formulations intended for intraluminal delivery must protect their payloads during GI transit and provide the required balance among mucus penetration, mucosal residence, and release in the target GI compartment [8, 29, 41]. Materials intended to remain at a lesion after endoscopic or other local administration must adhere to wet, continuously moving tissue while maintaining appropriate mechanical compliance and resistance to fluid washout [37]. Systemically administered carriers must remain stable in circulation and limit premature immune clearance. They may also need to cross the vascular wall and tissue interstitium to reach lesions and, when required, enter target cells [33]. A delivery system optimized for one administration route may not be directly applicable to another because each route presents distinct physiological barriers and corresponding material design requirements, as summarized in Fig. 2.

Some delivery systems combine local retention with controlled release or tissue protection with immune modulation. Adding more functions does not necessarily improve therapeutic performance. Each added function should address a specific delivery barrier or therapeutic objective and produce a measurable improvement in a relevant endpoint. Otherwise, an overly complex design can obscure which material property contributes to the therapeutic effect.

## Advanced biomedical materials and drug delivery system design for GI applications

The route- and region-specific barriers described above determine the functions required of GI biomaterials. This section considers three related aspects of advanced biomaterials: their therapeutic function, material or delivery-platform class, and targeting or activation strategy.

### Classification of advanced biomaterials for GI delivery

Advanced biomaterials for GI applications are grouped here into five broad functional categories: payload protection, regulation of mucosal residence and barrier transport, stimuli-responsive release, tissue regeneration and barrier repair, and immune or microbiome modulation. Many materials and formulations perform more than one function, so these categories are not mutually exclusive. For clarity, each example is discussed under the category that best reflects its intended GI use.

Payload-protective materials shield therapeutics from acid- or enzyme-mediated degradation until they reach the intended GI compartment. Examples include pH-dependent grades of Eudragit® and hydroxypropyl methylcellulose phthalate, which can limit premature release during gastric transit and permit release in higher-pH intestinal regions [8, 42, 43]. These materials must protect the payload during the relevant phase of GI transit while allowing its release at the target site.

Materials that regulate mucosal residence and barrier transport address two different delivery constraints. Rapid clearance shortens local residence, whereas excessive trapping in mucus hinders drug delivery to epithelial cells. Chitosan, alginate, hyaluronic acid, poly(acrylic acid)-based materials such as carbomers, and thiolated polymers have been investigated as mucoadhesive components in formulations intended to prolong mucosal residence, although their adhesive performance depends on polymer structure and formulation conditions [44, 45]. Their interactions with mucus may involve electrostatic attraction, hydrogen bonding, and interpenetration or entanglement of polymer and mucin chains [28]. Thiolated polymers can additionally form covalent disulfide bonds with cysteine-rich domains of mucins [45]. By contrast, when epithelial access is required, densely coating particles with low-molecular-weight polyethylene glycol (PEG) can reduce adhesive interactions with mucins and facilitate diffusion through mucus [28, 46]. Appropriately designed zwitterionic hydrogel coatings have also been used to promote mucus permeation in specific formulations [47]. Surface chemistry should be selected according to the delivery objective, either to increase mucoadhesion and prolong local residence or to reduce nonspecific interactions with mucus and facilitate epithelial access.

Stimuli-responsive materials use regional pH, enzymatic activity, redox conditions, or microbial metabolism to trigger changes in permeability, degradation, or payload release. Enteric polymers, azoreductase-cleavable linkages, and oxidation-sensitive bonds are representative examples [8, 38].

Materials for tissue regeneration and barrier repair are designed to support mucosal healing and barrier restoration. ECM-derived scaffolds, including small intestinal submucosa and decellularized gastric matrices, retain structural and biochemical cues that can support epithelial repair, neovascularization, and immune modulation in preclinical models [10, 48, 49]. Synthetic hydrogels can be functionalized with cell-adhesive peptides, and their mechanical properties and degradation rates can be adjusted [11, 50]. For materials intended for local GI repair, relevant design factors include wet-tissue adhesion or another means of local retention, mechanical compatibility, degradation, and host tissue remodeling [12, 13]. Drug-release behavior must also be considered when a therapeutic agent is incorporated [11].

Materials that modulate immune or microbial activity are designed to alter local inflammatory signaling, microbiota composition, or microbial metabolism while remaining compatible with the mucosa. Some cell membrane coatings can reduce macrophage uptake and immune clearance [51]. Selected ECM-derived matrices can modulate macrophage responses [48, 52]. In preclinical models, enteric polymer coatings and hydrogel microspheres have improved resistance to GI conditions and increased the intestinal accumulation or colonization of probiotic or engineered bacteria [53, 54]. Evaluation should distinguish intended local modulation from unintended inflammatory responses or adverse changes in microbiota composition or function.

### Drug delivery platforms for GI applications

Each therapeutic function can be achieved with more than one material class. The choice among hydrogels, lipid-based carriers, inorganic or polymeric nanoparticles, MOFs, cell membrane-coated carriers, and ECM-derived scaffolds depends on the administration route, required function, safety profile, and manufacturability.

Hydrogels are three-dimensional polymer networks that retain large amounts of water and can be designed with tunable mechanical properties. Their biocompatibility depends on polymer composition and crosslinking chemistry [11, 50]. Natural-polymer hydrogels, including those based on chitosan, alginate, hyaluronic acid, and gelatin, may be biodegradable. Their degradation behavior and mechanical properties depend on the polymer source, chemical composition, processing method, and crosslinking strategy [50, 55]. Synthetic hydrogels based on polyethylene glycol (PEG), poly(vinyl alcohol), or poly(N-isopropylacrylamide) allow greater control over composition and physicochemical properties; stimulus-responsive groups or bioactive cues can be introduced when required [11, 50]. Among these bioactive components, enzymes can be immobilized within hydrogel networks or used to mediate polymerization and crosslinking, thereby introducing catalytic or stimulus-responsive functions [56]. Interpenetrating polymer networks can combine complementary properties of natural and synthetic components, and three-dimensional printing can produce complex, cell-containing hydrogel structures [57, 58].

Lipid-based carriers, including liposomes, solid lipid nanoparticles, and nanostructured lipid carriers, can offer favorable biocompatibility and have established clinical precedents, particularly among liposomal products. Some manufacturing methods for solid lipid nanoparticles and nanostructured lipid carriers are amenable to scale-up, although this remains formulation-dependent [40]. For oral delivery, bile salts and digestive lipases can alter the structure and release behavior of lipid formulations [59]. Depending on the carrier, limitations may include premature drug leakage, low or variable loading of hydrophilic macromolecules, drug expulsion during lipid crystallization, and storage instability [60]. Surface modification and stimulus-responsive components can alter mucus interactions and release behavior, but their effects depend on the formulation [59, 60].

Inorganic nanoparticles used in GI applications include mesoporous silica nanoparticles, gold nanorods, and iron oxide nanoparticles. Their functions depend on composition. Mesoporous silica nanoparticles provide high surface areas and tunable pores for drug loading and controlled release; gold nanorods can support photoacoustic imaging and light-triggered photothermal treatment; and iron oxide nanoparticles provide magnetic responsiveness for magnetic resonance imaging and magnetic hyperthermia. These properties may also be integrated with drug delivery for combined diagnostic and therapeutic applications [61]. Their in vivo fate varies substantially among materials: some release ions or undergo chemical transformation, whereas others may persist as particles in tissues. Depending on composition and exposure, potential risks include ion toxicity, oxidative stress, tissue persistence, and slow clearance [33, 62]. Evaluation should consider how core composition, particle size, surface chemistry, administration route, and cumulative dose affect dissolution, clearance, biocompatibility, and long-term in vivo fate [33, 62].

Polymeric nanoparticles based on biodegradable polyesters, including poly(lactic-co-glycolic acid) (PLGA), polycaprolactone, and polylactic acid, can encapsulate different cargos and provide controlled release. Their loading and release profiles depend on the cargo, polymer, and fabrication method [33, 63]. For intraluminal GI delivery, unmodified polymeric particles may show limited mucosal residence or become trapped in mucus, depending on their surface chemistry [29]. Chitosan coatings can increase mucoadhesion, whereas dense hydrophilic coatings can reduce mucus binding, but these modifications introduce additional formulation variables that must be controlled during scale-up [29, 40].

MOFs are porous coordination materials with high specific surface areas and tunable pore sizes [64]. In the Pd(H)@ZIF-8 (zeolitic imidazolate framework-8) system developed for *H. pylori* treatment, gastric acid decomposed the particles and released Zn^2^⁺ and hydrogen [65]. Potential toxicity associated with the metal nodes or organic linkers and the reproducibility of synthesis at scale require formulation-specific assessment before translation [40, 64].

Cell membrane-coated carriers comprise synthetic nanoparticle cores coated with membranes derived from erythrocytes, macrophages, cancer cells, or platelets. The coating combines the drug-loading capacity of the synthetic core with membrane-mediated immune evasion, homotypic recognition, or cellular interactions [66, 67]. Most reported systems remain preclinical. Translation requires control of membrane sourcing, pathogen and endotoxin contamination, preservation of membrane proteins, batch-to-batch reproducibility, and storage stability [66].

ECM-derived materials, including decellularized matrices and ECM hydrogels, retain structural and biochemical components that can support tissue repair and modulate host responses [52, 68]. However, decellularization and subsequent processing can alter their composition, mechanical properties, and biological activity. Residual cellular material and DNA, source- and batch-dependent variability, sterilization-associated matrix damage, and xenogeneic antigens therefore require systematic control [68, 69].

No single material class is suitable for all GI indications; selection should reflect the administration route, dominant physiological barrier, required therapeutic function, and manufacturing constraints. For oral or intraluminal delivery, polymeric and lipid carriers offer versatile formulation options, but GI fluids can alter their stability and release, and their interactions with mucus require control. Hydrogels can support local residence and conformal coverage but can present problems related to swelling, bulk, and administration. ECM-derived materials can provide bioactive cues, but their biological origin complicates standardization. Inorganic nanoparticles and MOFs add optical, catalytic, or high-capacity loading functions but require closer evaluation of transformation products, residual particles, and cumulative exposure. Cell membrane-coated carriers are generally selected when immune evasion or source-cell-derived recognition is required, although membrane sourcing and quality control remain important constraints. The main functions, advantages, and limitations of these material classes and carrier architectures are compared in Table 1.

**Table 1:** Comparison of major biomaterial systems for gastrointestinal delivery and therapeutic intervention

Material system	Representative composition or architecture	GI-relevant functions and administration settings	Main advantages	Principal limitations	References
Hydrogels and adhesive polymer networks	Networks based on chitosan, alginate, hyaluronic acid, gelatin, polyethylene glycol, or poly(vinyl alcohol)	Local or endoscopic delivery; mucosal residence; sustained release; defect sealing; tissue repair	Conformal coverage; tunable mechanics; potential for wet-tissue adhesion; adaptable cargo incorporation	Swelling, bulkiness, and susceptibility to washout; formulation-dependent mechanical weakness; variability in natural polymers	[11, 50, 55]
Lipid-based carriers	Liposomes, solid lipid nanoparticles and nanostructured lipid carriers	Oral, local, or systemic delivery of small molecules and macromolecules	Generally favorable biocompatibility; adaptable formulation; established clinical precedents, particularly for liposomal products	Destabilization by bile salts or lipases; premature leakage; storage and cargo-loading limitations	[59, 60]
Inorganic nanoparticles	Mesoporous silica nanoparticles, gold nanorods, and iron oxide nanoparticles	Imaging; catalytic or photothermal treatment; magnetic guidance	Composition-dependent optical, magnetic, or catalytic functions	Composition-dependent ion toxicity, oxidative stress, tissue retention and uncertain clearance	[33, 61, 62]
Polymeric nanoparticles	Carriers based on poly(lactic-co-glycolic acid), polycaprolactone, polylactic acid, or chitosan	Cargo protection; controlled release; regulation of mucus interactions; oral or systemic delivery	Adjustable composition, drug loading, release and surface properties	Mucus trapping or inadequate retention, depending on surface chemistry; added quality-control variables after functionalization	[29, 33, 40, 63]
Metal–organic frameworks	Zeolitic imidazolate framework-8 and related porous coordination materials	High-capacity loading; pH-responsive release; co-delivery	Tunable pore structure and composition	Potential exposure to metal ions or organic linkers; formulation-dependent disassembly and in vivo fate; synthesis and quality-control complexity	[40, 64, 65]
Cell membrane-coated carriers	Nanoparticle cores coated with erythrocyte, macrophage, platelet or tumor-cell membranes	Mainly systemic delivery requiring immune evasion or biological recognition	Retention of selected membrane-associated biological functions	Membrane-source variability; pathogen and endotoxin control; protein integrity; batch-to-batch consistency and storage stability	[51, 66, 67]
Extracellular matrix-derived materials	Decellularized tissues, extracellular matrix hydrogels, and materials derived from small intestinal submucosa	Local barrier repair and tissue regeneration	Native-like structural and biochemical cues	Residual DNA or cellular components; source variability; limited mechanical control; sterilization and animal-source concerns	[52, 68, 69]

Biomaterials are classified in the text by their predominant therapeutic functions, whereas this table compares material and delivery-platform classes; these categories are complementary rather than mutually exclusive

*Abbreviations*: *GI* gastrointestinal, *ZIF-8* zeolitic imidazolate framework-8

### Material-integrated targeting strategies

Material-integrated targeting can rely on passive biodistribution or physiological uptake, ligand-mediated recognition, or site-selective activation by local stimuli. The choice of mechanism depends on the administration route and target compartment.

Passive targeting uses the intrinsic biodistribution or physiological uptake of a carrier. Systemically administered tumor nanocarriers may accumulate in lesions with increased vascular permeability through the EPR effect, whereas orally administered particles may be taken up by microfold cells overlying Peyer’s patches [70, 71]. Passive targeting does not ensure lesion-specific delivery and is influenced by carrier size, shape, and surface properties [33, 72]. The heterogeneity of the tumor microenvironment can also contribute to variable nanoparticle delivery [71]. Particle accumulation in animal models does not by itself establish clinical efficacy; lesion exposure and therapeutic response require direct assessment [33, 71].

Active targeting uses ligand-receptor interactions to enhance cellular binding and uptake at target sites [73]. Reported targeting ligands include lectins, antibodies, peptides, and receptor-binding small molecules [8, 74]. Selection depends on target expression, binding affinity, and potential immunogenicity. Active ligands can improve receptor-mediated uptake but do not necessarily increase total lesion exposure. For systemically administered carriers, vascular extravasation and interstitial transport remain limiting; for oral carriers, mucus transport and epithelial access may remain limiting. Increasing ligand density alone may not compensate for these upstream barriers [29, 33, 74].

Stimulus-responsive release can contribute to targeting when the activating condition differs sufficiently between the intended site and surrounding compartments to improve spatial selectivity. Regional pH changes can enable region-dependent release [8]. Bacterial azoreductase activity can support colon-dependent release, whereas sufficiently elevated matrix metalloproteinase (MMP) activity or reactive oxygen species (ROS) can support disease-associated release in inflamed tissue [36, 75, 76]. If a trigger changes the release rate without favoring the intended site, the formulation is better described as stimulus-responsive rather than targeted.

Multiple targeting elements can be combined when a physiological cue is insufficiently selective. For example, sequential pH- and enzyme-responsive elements have been combined so that an outer pH-responsive matrix protects the formulation during GI transit and an enzyme-responsive inner component releases the drug at inflamed intestinal sites [75]. Each additional targeting element should produce a measurable improvement in site-specific delivery or therapeutic effect that justifies the added manufacturing and safety requirements.

## Therapeutic applications of advanced materials in gastrointestinal diseases and gut-associated metabolic disorders

Each GI disorder places different demands on biomaterials, depending on the target compartment and administration route. The following discussion examines how cargo protection, local residence, site-selective release, barrier transport, and tissue repair have been applied in PUD and *H. pylori* infection, GI perforation, IBD, GI malignancies, and gut-associated metabolic disorders [8, 12], with attention to disease-specific outcomes and the available preclinical or clinical evidence.

### Peptic ulcer disease

PUD comprises mucosal defects in the stomach or proximal duodenum that extend into the submucosa or deeper, distinguishing ulcers from superficial erosions [77]. Ulcer formation results from an imbalance between mucosal protective mechanisms and aggressive factors [77, 78]. *H. pylori* infection and the use of nonsteroidal anti-inflammatory drugs (NSAIDs) are well-established risk factors for PUD [77, 79]. Clinical management depends on the cause and complications and commonly includes *H. pylori* eradication, acid suppression, and withdrawal or reduction of ulcerogenic NSAIDs [77]. Biomaterial strategies for PUD aim to support *H. pylori* eradication or protect and repair injured gastric mucosa.

#### Eradication of *H. pylori*

*H. pylori* is a Gram-negative bacterium that colonizes the gastric mucosa. It is a major cause of PUD and a recognized risk factor for gastric cancer [80]. It produces urease, which hydrolyzes urea to generate ammonia and helps buffer its acidic surroundings. Bacterial virulence factors and the host inflammatory response contribute to gastric mucosal injury [81]. Current eradication regimens combine an acid suppressant, such as a proton pump inhibitor or potassium-competitive acid blocker, with one or more antibiotics and, in some regimens, bismuth [82]. Eradication success varies because of antibiotic resistance, incomplete adherence, prior antibiotic exposure, and regimen-related factors; multidrug therapy can also cause gastrointestinal adverse effects [82, 83]. These limitations have prompted the investigation of more localized or antibiotic-sparing approaches, although the available biomaterial strategies remain preclinical. These strategies can be grouped according to three main functions: receptor-mimetic bacterial capture, protection or immobilization of antimicrobial agents under gastric conditions, and microenvironment-responsive or externally activated antibacterial treatment.

Receptor-mimetic capture offers a nonbactericidal approach to limiting bacterial adhesion [84]. Gonçalves et al. [85] developed glycan-coated chitosan microspheres to capture *H. pylori* through adhesin-glycan interactions. Lewis b (Leᵇ) and sialyl-Lewis x (sLeˣ) glycans were immobilized on the chitosan microspheres using azide-alkyne click chemistry. Coupling through the polymer’s primary hydroxyl groups preserved the primary amine groups that contribute to mucoadhesion. These functionalized microspheres, Leᵇ-Mic and sLeˣ-Mic, mimicked host glycan receptors and preferentially bound *H. pylori* strains expressing the corresponding adhesins, BabA or SabA, in vitro. In tissue experiments, Leᵇ-Mic reduced adhesion of BabA-positive *H. pylori* in mouse and human gastric sections in vitro and in freshly excised mouse stomachs ex vivo. The study therefore established proof of concept for glycan-mediated bacterial capture but did not test eradication in an infected animal model or demonstrate clearance of captured bacteria through the intestine.

A second approach is to protect antimicrobial agents and maintain their local activity under acidic, mucus-covered gastric conditions. Deng et al. [86] engineered CaCO_3_@Fe-TP@EggPC nanoliposomes (CTE NLs). The particles comprised a calcium carbonate core coated with an Fe^3^⁺-tea polyphenol complex and an outer egg phosphatidylcholine layer. In simulated gastric fluid and mice, calcium carbonate consumed protons and temporarily increased gastric pH, thereby limiting acid-induced dissociation of the Fe-tea polyphenol complex. Fluorescence imaging showed that the particles entered and remained within gastric mucus, while in vitro imaging was consistent with fusion of the phospholipid layer with, and disruption of, the bacterial membrane. In the reported mouse experiment, bacterial burdens were 6.36 × 10^7 CFU/g in the PBS group and 4.43 × 10^6 CFU/g in the CTE NL group; alpha-diversity indices did not differ significantly among groups. These findings support acid neutralization, antibacterial activity, and short-term modulation of selected microbiome features in mice but do not establish restoration of the intestinal microbiome in patients.

The need to maintain antimicrobial activity under gastric conditions also applies to antimicrobial peptides (AMPs). MSI-78 A (pexiganan A) is among the few AMPs reported to be active against *H. pylori* [87, 88]. Free MSI-78 A may require relatively high concentrations because of self-aggregation and proteolytic degradation. Fonseca et al. [88] used chitosan with a low degree of acetylation (6.6 ± 1.2%), whose primary amines contribute to gastric mucoadhesion, to prepare chitosan microspheres (ChMic) by spray drying. Partial genipin crosslinking before spray drying prevented aggregation of the small microspheres, which had a mean diameter of approximately 4 μm, close to the length of *H. pylori*. Their small size also increased the surface-area-to-mass ratio available for AMP grafting. A chemoselective thiol-maleimide coupling reaction with a PEG_113_ spacer was then used to immobilize MSI-78 A on ChMic (AMP-ChMic). The authors proposed that immobilization limited peptide self-aggregation, while microscopy showed close microsphere-bacterium contact and bacterial membrane disruption. After preincubation in simulated gastric fluid or with pepsin, AMP-ChMic retained bactericidal activity against the *H. pylori* J99 strain. In growth medium, grafted MSI-78 A was bactericidal at an estimated peptide concentration of 277 μg/mL, whereas free MSI-78 A had a minimum bactericidal concentration of 512 μg/mL. However, these experiments were conducted in vitro, and grafting did not completely prevent proteolytic degradation. CTE NLs and AMP-ChMic use different mechanisms, but both are intended to maintain effective local antimicrobial exposure under gastric conditions.

A third approach uses microenvironment-responsive or externally activated mechanisms to generate local antibacterial effects. Zhang et al. [65] engineered a multistage, microenvironment-responsive platform by integrating hydrogen-producing Pd(H)@ZIF-8 nanoparticles into an ascorbate palmitate (AP) hydrogel matrix. The negatively charged AP hydrogel was designed to adhere preferentially to positively charged inflamed gastric mucosa and release the nanoparticles following matrix metalloproteinase-associated hydrolysis. Collagenase IV-responsive release was demonstrated in vitro, and greater gastric retention was observed in infected than in healthy mice. The released Pd(H)@ZIF-8 nanoparticles subsequently disassembled in acidic gastric fluid, releasing Zn^2^⁺ from ZIF-8 and H_2_ from hydrogen-loaded Pd nanoparticles. In vitro assays showed increased bacterial-membrane permeability and protein leakage and reduced urease activity after Pd(H)@ZIF-8 treatment. In infected mice, the complete Pd(H)@ZIF-8@AP formulation reduced gastric bacterial burden and markers of inflammation and oxidative stress while improving markers of mucosal repair. These findings support combined antibacterial and mucosal-protective activity, although the separate in vivo contributions of H_2_ and Zn^2^⁺ were not established.

Liu et al. [89] developed a sonodynamic system consisting of verteporfin-loaded poly(lactic-co-glycolic acid) nanoparticles coated with a lecithin bilayer (Ver-PLGA@Lecithin). In vitro, lecithin-coated PLGA particles adsorbed vacuolating cytotoxin A (VacA) from H. pylori culture supernatant through protein-corona formation. Treatment with these particles reduced the cytotoxicity of the supernatant toward AGS gastric epithelial cells even without ultrasound. Ultrasound exposure at 0.5 W cm⁻^2^ for 10 min induced reactive oxygen species generation, identified predominantly as singlet oxygen in vitro, and killed H. pylori. In female mice with gastric H. pylori infection, four oral treatment cycles at 48-h intervals, each followed by localized ultrasound applied to the skin overlying the stomach, reduced gastric bacterial burden by approximately 50-fold relative to the vehicle control and produced an effect comparable to standard triple therapy under the study conditions.

These three groups address different barriers to *H. pylori* treatment and should not be viewed as interchangeable. Receptor-mimetic microspheres reduce bacterial adhesion without direct bactericidal pressure, but their performance may depend on the adhesins expressed by individual bacterial strains. Acid-neutralizing and peptide-conjugated carriers are intended to preserve antimicrobial activity in the acidic, mucus-covered, and protease-rich stomach. Microenvironment-responsive and ultrasound-activated systems generate local antibacterial effects and may also address inflammation, toxin activity, or mucosal injury. All of these approaches remain preclinical, with evidence derived from in vitro, ex vivo, or animal studies. Across these approaches, reproducible gastric residence, activity against heterogeneous *H. pylori* strains, and effects on the gastric mucosa and intestinal microbiota require further investigation. Inorganic systems additionally require characterization of component transformation and clearance. Peptide-conjugated systems require in vivo confirmation of peptide stability and efficacy, whereas sonodynamic treatment requires clinically relevant assessment of ultrasound penetration, dosimetry, the timing between oral dosing and ultrasound exposure, and repeated-use safety.

#### Enhancement of the gastric mucosal protective barrier

The gastric mucosa is protected by the mucus-bicarbonate layer, epithelial tight junctions, adequate mucosal blood flow, and rapid epithelial restitution and renewal [78, 90]. These defenses limit the penetration of acid and pepsin and help the mucosa withstand luminal irritants while maintaining local homeostasis [78]. When protective factors such as prostaglandins, growth factors, and antioxidant systems are overwhelmed by damaging factors, including *H. pylori* infection, NSAID-mediated suppression of prostaglandin synthesis, excessive acid-peptic activity, ischemia during severe physiological stress, ethanol, and bile reflux, mucosal defense is weakened [78, 90]. Depending on the cause, inflammation and oxidative stress can disrupt epithelial integrity, weaken mucus protection, increase reactive oxygen species (ROS), and promote cell death, thereby contributing to erosion or ulceration [90–92]. Restoring mucosal integrity is therefore an important therapeutic goal in gastric ulcer repair.

Materials for gastric mucosal repair must remain at the lesion, protect the wound, and support tissue regeneration. Relevant design requirements include route-appropriate biocompatibility, wet-tissue adhesion, mechanical compliance with gastric motility, and predictable degradation or elimination [93]. Decellularized extracellular matrix (ECM) scaffolds can provide cell-adhesive and regenerative cues and, in some settings, promote a pro-remodeling macrophage response [52]. Ni et al. [48] prepared porcine gastric extracellular matrix (gECM) using a hypertonic salt solution and enzymatic treatment, combined it with high-molecular-weight hyaluronic acid (HA) and omeprazole, and freeze-dried the composite to produce a hyaluronic acid/gastric ECM (gHECM) patch. This elastic composite was designed to accommodate gastric motion and limit postoperative adhesion to surrounding organs, although anti-adhesion was not directly quantified. In the rat gastric-wall-defect model, gHECM was associated with increased M2 macrophages, less persistent inflammatory infiltration, and faster muscle and tissue repair than polypropylene patch.

Gastric adhesives must maintain wet-tissue adhesion under acidic and mechanically dynamic conditions. Xie et al. [94] developed a gastric-adhesive hydrogel using ionized N-acryloyl phenylalanine (iAPA) and N-[tris(hydroxymethyl)methyl] acrylamide (THMA) as monomers. Under acidic gastric conditions, iAPA undergoes a hydrophilic-to-hydrophobic transition and forms a network through phenyl-assisted multivalent hydrogen bonding, while THMA hydroxymethyl groups form hydrogen-bonding clusters within the network and noncovalent interactions with functional groups on the tissue surface. This mechanism generated an acid-stable barrier with a reported wet adhesion strength of 33.4 kPa to gastric tissue and structural stability for 14 days in simulated gastric fluid. In an acetic acid-induced rat ulcer model, the hydrogel accelerated healing while reducing inflammation and promoting capillary formation.

To reconcile endoscopic deliverability with prolonged gastric retention, Liu et al. [13] copolymerized β-carboxylic-amide-functionalized N-acryloyl phenylalanine (NA-Mal) with hydroxyethyl acrylamide (HEAA) to obtain the sequentially pH-responsive polymer adhesive PNMH. Electrostatic repulsion between the ionized carboxylate groups maintained a low-viscosity precursor suitable for delivery through an endoscopic catheter. Upon exposure to gastric acid, carboxylate protonation induced hydrophobicity-assisted hydrogen bonding, resulting in gelation within approximately 4 s and immediate wet-tissue adhesion of approximately 24.1 kPa. Subsequent hydrolysis of the β-carboxylic amide shifted the dominant interactions toward hydrophobicity-assisted electrostatic and cation-π interactions. The adhesion strength increased to 34.3 kPa at 6 h, and hydrogel-tissue integration was maintained for 30 days under simulated gastric conditions. PNMH remained at the rat gastric site for at least 96 h and blocked 83% of pepsin transport in vitro. In an acetic acid-induced rat ulcer model, a single local application reduced ulcer area to 2.8 mm^2^ on day 7, compared with 20.7 mm^2^ in the saline group and 14.0 mm^2^ in the sucralfate group; complete healing was observed by day 14. In a porcine model in which gastric ulcers were created by endoscopic submucosal dissection, endoscopically delivered PNMH increased the day-10 closure ratio to 92.1%, versus 79.2% and 73.4% in the saline and sucralfate groups, respectively, while reducing inflammatory infiltration and promoting collagen deposition and epithelial regeneration.

In acidic gastric fluid, hydrogen bonding within starch-based dressings is weakened, which reduces gel stability and tissue adhesion. Liu et al. [95] mixed polyvinylpyrrolidone (PVP)-modified magnesium diboride (MgB_2_) nanoparticles (MB NPs) with starch to form a hybrid powder (MBSG). Upon contact with gastric acid, MBSG formed an in situ gel layer within approximately 20 min that adhered to the ulcerated mucosa. The acid-responsive decomposition of MgB_2_ nanoparticles releases hydroxide ions, forming a local alkaline gradient. This process enhances the electron density of hydrogen-bond acceptors within the gel, strengthening the hydrogen-bonded network, and reduces ion–dipole interactions between the gel’s hydroxyl groups and gastric acid, thereby improving tissue adhesion. The resulting MBSG dressing showed an adhesive force 3.96-fold higher than that of clinical colloidal bismuth pectin in simulated gastric juice. It also neutralized excess H⁺ and showed concentration-dependent ROS-scavenging activity, including approximately 92% scavenging of 2 mM H_2_O_2_ at an MBSG concentration of 0.05 mg mL⁻^1^. In an ethanol-induced acute gastric-ulcer model in mice, MBSG reduced M1 macrophage activation and apoptosis, covered the ulcer bed, and was associated with increased CD31 staining. The formulation released Mg^2^⁺ under acidic conditions, but the contribution of Mg^2^⁺ to angiogenesis was not isolated experimentally.

To address the weak adhesion, excessive swelling, and limited antioxidant capacity of conventional hydrogels in the dynamic acidic gastric environment, Ni et al. [96] grafted lactobionic acid (LA) onto chitosan (CS) through amide formation to obtain the material described as lactose-modified chitosan (CSLA). They then combined CSLA with polyethylenimine (PEI) and poly(acrylic acid) (PAA), followed by lyophilization and grinding to produce a PPCL bioadhesive powder. Upon contact with water, the powder formed a physically crosslinked hydrogel within 3 s through hydrogen bonding and electrostatic interactions. The resulting hydrogel showed an adhesion strength greater than 20 kPa on ex vivo porcine gastric tissue, a low swelling rate, resistance to simulated gastric fluid, and broad ROS-scavenging activity, allowing it to form a protective barrier against gastric acid and digestive enzymes. In an acetic acid-induced rat gastric-ulcer model, PPCL reduced oxidative stress and inflammation, promoted reepithelialization, angiogenesis, and mucosal repair, and produced a smaller ulcer index than aluminum phosphate gel, the commercial comparator used in the study, on day 14.

Bleeding gastric ulcers require materials that provide rapid hemostasis while remaining compatible with subsequent mucosal repair. Wen et al. [97] developed carboxymethyl chitosan (CMC) hydrogels. CMC was dynamically crosslinked through Schiff-base reactions with either aldehyde-functionalized xanthan gum (XG-CHO) or oxidized konjac glucomannan (OKGM), while incorporating the thrombin-derived peptide TCP-25. The dynamic imine bonds provided self-healing properties. Both formulations, TCP-CX and TCP-CO, gelled within 8–9 s, and their injectability was demonstrated in vitro; endoscopic delivery was not tested. In a rat liver-laceration model, TCP-CX achieved hemostasis in approximately 9 s and reduced blood loss from 1961 ± 5 to 145 ± 5 μL, whereas TCP-CO achieved hemostasis in approximately 16 s. In an ethanol-induced bleeding gastric-ulcer model, treatment was repeated every 6 h, and the reported 24-h ulcer-inhibition ratios were 92% for TCP-CX and 90% for TCP-CO. The porous structure had mean pore diameters of approximately 90 and 120 μm for TCP-CX and TCP-CO, respectively, and was proposed to retain blood and coagulation components while TCP-25 promoted coagulation. Dynamic covalent C = N bonds formed through Schiff-base reactions produced pH-dependent release. At 48 h, cumulative TCP-25 release at pH 2.0 was approximately 51% from TCP-CX and 65% from TCP-CO, compared with approximately 81% from both hydrogels at pH 7.4. In addition, both formulations showed antibacterial activity against standard strains and several multidrug-resistant clinical isolates and maintained greater than 90% NIH-3T3 cell viability under the tested conditions. The reported lap-shear adhesion strengths of 20 kPa for TCP-CX and 27 kPa for TCP-CO were measured on wet porcine skin rather than gastric tissue. Partial adhesion remained after 48 h of immersion in simulated gastric fluid at pH 2.0, but resistance to gastric peristalsis was not tested.

For nonbleeding ulcers, prolonged gastric residence may improve the local effects of materials designed to reduce oxidative stress and support mucosal repair. Zheng et al. [98] obtained a homogeneous Astragalus polysaccharide, APS13, through purification and structural characterization. Using heat-induced self-assembly, they complexed APS13 with glycyrrhizic acid (GA) to form the GA-APS hydrogel. This process required no chemical crosslinkers, forming a fibrous micellar network with an average pore diameter of 15 μm through intermolecular noncovalent interactions. The hydrogel maintained adhesion for 60 min to ex vivo porcine gastric mucosa immersed in simulated gastric fluid at pH 2.0. After oral administration to rats, GA-APS remained in the stomach for approximately 2 h, whereas GA and APS alone were largely cleared within 1 h. In vitro release of the hydrogel components continued for nearly 6 h under acidic conditions and for more than 10 h under neutral conditions. Its effects were evaluated in a water-immersion-stress model of acute gastric ulcer in rats. GA-APS reduced ulcer area and tissue ROS levels and lowered the expression of apoptosis-inducing factor and cleaved caspase −3. In H_2_O_2_-treated gastric epithelial cells and gastric organoids, it also preserved mitochondrial membrane potential and reduced apoptosis. These findings associate the therapeutic effect with longer gastric residence and attenuation of ROS-related apoptosis but do not establish efficacy in chronic ulcer disease.

These studies support distinct choices for gastric mucosal repair. Decellularized ECM patches were investigated for regenerative support and immunomodulation, whereas in situ hydrogels and powders prioritize wet-tissue adhesion, hemostasis, and local delivery. The appropriate material format therefore depends on whether the lesion is bleeding, requires mechanical sealing, or primarily needs sustained mucosal protection. Direct efficacy comparisons are limited because these studies used different rodent injury models, comparators, treatment schedules, and endpoints. All of these systems remain preclinical; further development will require reproducible delivery, durable adhesion without impaired gastric motility, predictable degradation or elimination, and broader validation in clinically relevant large-animal models.

### Gastrointestinal perforation

Gastrointestinal perforation is a serious GI emergency characterized by a full-thickness defect in the digestive tract wall, which allows luminal gas or contents to enter the surrounding body cavity. It may arise from peptic ulceration, inflammation, malignancy, trauma, or iatrogenic injury during endoscopic procedures [99, 100]. Management depends on the location and size of the defect, the degree of contamination, the time of recognition, and the patient’s clinical condition. Selected iatrogenic perforations can be closed endoscopically, whereas surgery remains necessary when endoscopic closure is not feasible or fails, or when the patient develops clinical deterioration or peritonitis [99, 101]. Available endoscopic methods include clips, suturing devices, covered stents, and endoscopic vacuum therapy. However, no single method is suitable for every defect, and selection depends on the defect characteristics and available expertise [99, 101]. These limitations motivate the development of endoscopically deliverable materials that can provide hemostasis, seal the defect, deliver therapeutics, and support healing. Adhesive hydrogels and asymmetric patches have been investigated, mainly in preclinical models. Hydrogels can provide conformal coverage and wet-tissue adhesion, whereas asymmetric Janus patches can combine a tissue-facing adhesive surface with a non-adhesive outward surface to reduce unintended attachment to adjacent organs.

#### Materials for sealing and promoting healing

Conventional surgical and endoscopic closure methods remain essential. Biomaterials intended to supplement these methods must form a fluid-tight seal on wet tissue, accommodate tissue deformation, resist washout, and retain sufficient mechanical integrity during healing [102]. Their degradation should also be predictable. Drug loading and pro-healing activity may be useful in some applications but are not required for every sealant. Different types of adhesives have distinct advantages and limitations: cyanoacrylates polymerize rapidly and form strong tissue bonds, but heat generated during polymerization and the potential toxicity of degradation products can limit their use. Fibrin sealants, by contrast, are biodegradable and generally induce little inflammation but provide only weak adhesion [103]. Material requirements should therefore be defined according to the defect and intended administration route.

Studies of mucosal defects created by endoscopic submucosal dissection (ESD) provide useful information about gastric tissue adhesion and wound healing, although these models should not be treated as equivalent to full-thickness perforation. Nishiguchi et al. [104] prepared sprayable hydrophobically modified gelatin microparticles by introducing aliphatic side chains, followed by spray drying and thermal crosslinking. On contact with wound exudate, the particles swelled and assembled into an adherent hydrogel layer. Alkyl-chain length influenced both adhesion and cellular responses. The C8-modified formulation showed the highest ex vivo tissue adhesion among the C4, C6, and C8 formulations, but the C6 formulation was selected for animal testing because the more hydrophobic C8 and C12 formulations increased inflammatory cytokine expression and reduced cell proliferation in vitro. In a swine gastric ESD model, the C6-modified particles reduced inflammatory-cell infiltration and fibrotic markers and increased vascular staining at day 14 compared with untreated wounds. These findings support gastric mucosal wound protection and healing but do not demonstrate closure of a full-thickness perforation.

He et al. [105] developed an injectable, pH-responsive, self-healing hydrogel based on acryloyl-6-aminocaproic acid, denoted AA by the authors, and its N-hydroxysuccinimide derivative (AA-NHS). Dynamic hydrogen bonding supported self-healing, whereas the NHS esters reacted with primary amino groups on the tissue surface to strengthen wet adhesion. The hydrogel was delivered through a gastroscope and evaluated in swine gastric hemorrhage and post-ESD wound models. It controlled acute bleeding and reduced delayed rebleeding. During 28 days of follow-up, hydrogel-treated wounds showed fewer inflammatory cells and lower fibrotic-marker staining, together with greater vascular staining, than proton pump inhibitor-treated or untreated wounds. This study provides large-animal evidence for endoscopic hemostasis and mucosal wound healing, but not for full-thickness perforation closure.

Because in situ gels may show insufficient adhesion and lose their payload in dynamic internal environments, Xu et al. [106] used thiourea-catechol coupling to prepare hyaluronic acid-catechol-thiourea hydrogels that gelled within 5 s under the tested pH conditions. Spraying an oxidant onto the precursor solution enabled in situ formation and endoscopic delivery. In pigs, the hydrogel remained at gastric ulcer sites for at least 48 h. In rodent and porcine gastric ulcer models, treatment reduced inflammation and promoted re-epithelialization and angiogenesis. This work likewise concerns mucosal ulcer healing rather than full-thickness perforation closure.

To address acid-induced swelling and loss of adhesion in hydrogel sealants, Chen et al. [107] developed an acid-tolerant hydrogel bioadhesive (ATGel). They prepared a poly(HEMA-NVP) substrate by ultraviolet (UV) copolymerizing HEMA and NVP with PEGDA as a crosslinker and grafted poly(acrylic acid-co–N-hydroxysuccinimide acrylate ester) [poly(AA-NHS)] adhesive brushes onto its surfaces. The brush layer promoted wet-tissue adhesion through noncovalent interactions and covalent coupling between NHS ester groups and tissue amines. The ATGel maintained its mechanical and adhesive performance during 240 h of exposure to simulated gastric fluid at pH 2.0, sealed a 5-cm ex vivo porcine gastric incision without leakage for 48 h, and rapidly sealed 5-mm gastric perforations in rats. In the rat model, the hydrogel remained adherent at day 14, and both the ATGel and suture groups showed complete repair by day 28. Compared with sutures, ATGel treatment was associated with reduced inflammation and fibrosis and increased angiogenesis. In a separate rat subcutaneous implantation study, less than 10% of the initial material mass remained after 56 days.

Enhancing wet-tissue adhesion remains a critical challenge in the development of perforation-sealing biomaterials. Current hydrogel design strategies primarily follow two approaches: adhering prefabricated adhesive hydrogels [108, 109] on wet tissues and forming adhesive hydrogels on wet tissues in situ from precursor solutions [110–113]. The former strategy can be limited by restricted polymer diffusion and incomplete tissue contact, whereas rapid gelation of in situ systems may shorten the time available for interfacial bonding. Both limitations are related to restricted polymer mobility at the tissue–hydrogel interface or within the forming network. Peng et al. [114] mixed equal volumes of PEI and PAA solutions to form a self-healing polyelectrolyte complex (PEC). After freeze-drying and milling, they obtained a self-setting PEI/PAA adhesive powder. When exposed to water or phosphate-buffered saline (PBS), the PEC powder rapidly swelled to form microgels. On contact, the swollen microgels self-healed into a weakly crosslinked, cohesive hydrogel, with gel formation and initial adhesion completed within 2 s. Additionally, the physically crosslinked polymers diffused into the substrate polymer network, thereby enhancing adhesion to wet tissues such as chicken skin, pig heart, pig stomach, and intestinal mucosa. In vitro, the powder sealed defects in porcine stomach and small intestine. In a rat gastric-perforation model, it reduced wound area and supported complete mucosal bridging by day 7. The powder also conformed to irregular defects and could be applied using assistive devices. After implantation on the rat gastric serosa, the material remained visible during the 30-day observation period; histology showed mild chronic inflammation and fibrous encapsulation but no marked tissue necrosis. These findings support its dual role as a sealant and physical barrier in the tested preclinical models.

To further enhance the mechanical properties and tissue integration required for gastrointestinal repair, Chen et al. [115] developed an adhesive hydrogel with an interpenetrating polymer network (IPN). A bioactive gelatin network and a dynamically crosslinked poly(N-[tris(hydroxymethyl)methyl] acrylamide) (PTH) network enabled rapid in situ gelation, dissipated mechanical energy, and formed hydrogen and covalent bonds with gastric mucosa. In a mouse gastric-perforation model, the hydrogel plug sealed the defect and supported mucosal regeneration.

Focusing on the prevention of delayed postoperative complications, Munoz Taboada et al. [116] developed a sprayable hydrogel sealant. The system consists of polyethyleneimine-modified Pluronic micelles (PluPEI) and oxidized dextran. At 37 °C, concentrated formulations gelled in less than 2 s through reactions between micellar amines and dextran aldehydes. The precursor viscosities remained below 20 mPa · s, allowing delivery through a catheter passed through a commercial endoscope. In vitro, PEI groups buffered acid in a transwell system, and both fresh sealant and sealant preincubated for 7 days in simulated gastric fluid blocked more than 98% of pepsin. In pigs, endoscopic spraying covered bleeding gastric lesions during the 6-h observation period. Material was present in all treated nonperforated colonic lesions on day 3 but was not detected on days 7 or 14. In a separate model of clipped porcine colonic perforations, material remained at 33% of lesions on day 7. The study demonstrated endoscopic feasibility and short-term wound shielding but did not establish a significant reduction in postoperative complications.

A common strategy for experimental injectable hydrogel dressings is to mix two polymer precursors that form covalent crosslinks in situ, including imine (Schiff-base) formation and thiol–Michael addition. However, stability under gastric conditions depends on the crosslinking chemistry. Certain dynamic imine- and benzoxaborole-based networks are pH-responsive, and their mechanical integrity may change with pH [117, 118]. For gastric applications, stability should therefore be evaluated directly in acidic gastric fluid; loss of network integrity may compromise wound coverage. To improve hydrogel stability in the acidic gastric environment, Wang et al. [119] developed an ABA triblock copolymer poly[(N-isopropylacrylamide)-co-(N-acryloyl 6-aminocaproic acid)]-b-poly(ethylene glycol)-b-poly[(N-isopropylacrylamide)-co-(N-acryloyl 6-aminocaproic acid)] (ANGNA), synthesized via reversible addition-fragmentation chain transfer (RAFT) polymerization. The copolymer contained a central hydrophilic PEG block and terminal thermoresponsive P(NIPAM-co-A6ACA) blocks. Under gastric conditions, it underwent a rapid sol–gel transition and formed a physical hydrogel by supramolecular self-assembly. Hydrogen bonding and hydrophobic interactions between the terminal blocks allowed the hydrogel to self-heal after damage. The hydrogel was injectable and moldable and showed PEG-associated resistance to biofouling. In a rat model of gastric perforation, the hydrogel dressing simplified the procedure compared with conventional omentum patching and was associated with less postoperative adhesion and inflammation and greater early vascular density.

To combine mechanical closure with local regulation of the healing environment, Liu et al. [120] developed a mushroom-cap-inspired hyperboloid occlusion device based on a tough, acid-resistant double-network hydrogel. The network was formed from dimethylacrylamide (DMA) crosslinked with biodegradable N,N′-cystaminebis(acrylamide) (CBA) and sodium alginate (SA) ionically crosslinked by Ca^2^⁺. The hydrogel sheets were rolled into a multilayer, onion-like structure that could be compressed for endoscopic delivery and re-expanded at the perforation site. Silica nanoparticles (SNPs) were coated on the outer surface to strengthen rapid adhesion to the gastric wall. To suppress gastric acid and promote tissue repair, the device was loaded with the potassium-competitive acid blocker vonoprazan fumarate (VF) and acidic fibroblast growth factor (aFGF), respectively. In rabbits, the perforations and devices were created and placed surgically, although healing was subsequently monitored by gastroscopy. A separate minipig feasibility experiment demonstrated transesophageal endoscopic delivery to a 10-mm gastric perforation, expansion within 5 min, and continued occlusion at 96 h. During a 70-h recording, the VF group had intragastric pH above 4 for 83% of the time; at 96 h, the intragastric end had broken while the remaining hydrogel was still adhered to the perforation site. However, only one minipig was included in each treatment group. The aFGF-loaded device was associated with greater cell proliferation, mucosal repair, and vascular staining in rabbit experiments. Subcutaneously implanted hydrogels in rats decreased in size but remained detectable after 8 weeks, so complete degradation at the gastric repair site was not demonstrated. These findings provide preclinical proof of concept for endoscopic deployment and combined mechanical and pharmacological treatment, but the small animal numbers limit conclusions about efficacy and long-term safety.

Overall, these studies use three main approaches: interfacial adhesion, in situ formation of a conformal seal, and mechanical occlusion combined with local drug release. However, the evidence remains preclinical. Most full-thickness perforation studies used rodents and open surgical exposure, whereas large-animal endoscopic evidence remains limited. Findings from ESD wounds and gastric ulcers can inform material adhesion and healing but cannot substitute for direct testing in full-thickness perforation models. Further studies should evaluate endoscopic deployment, sealing under sustained gastrointestinal motion and fluid exposure, infection and leakage, degradation at the repair site, and comparison with clinically used closure methods [107, 114–116, 120].

#### Anti-adhesion materials

Postoperative adhesions are an important complication of abdominal and pelvic surgery [121]. Conventional bioadhesives may bond to both the defect and adjacent tissues, which can increase the risk of postoperative adhesions [122]. This problem has motivated the development of Janus bioadhesives. These materials feature an asymmetric architecture: one surface bonds to wet injured tissue, whereas the opposite surface remains non-adhesive and separates the repair from adjacent organs [122]. This spatial division allows the same patch to seal the defect while limiting contact-induced postoperative adhesion.

To seal gastrointestinal defects while limiting postoperative adhesion, Cui et al. [123] developed a Janus hydrogel by creating a complexation gradient within a poly(N-acryloyl-2-glycine) (PACG) network using cationic chitooligosaccharides (COS). The lightly complexed surface retained free carboxyl groups that could interact with tissue, while phase separation near this surface increased hydrophobicity and promoted interfacial water drainage. The reported underwater interfacial toughness was 149 and 144 J m⁻^2^ on porcine stomach and intestine, respectively. The carboxyl groups on the anti-adhesive surface were more extensively complexed and completely neutralized, making this surface non-adhesive and reducing postoperative tissue adhesion. In a rabbit gastric-perforation model, the preformed patch was pressed over a 0.5-cm defect for 30 s and remained attached without suturing. No gross adhesion to adjacent tissue was observed at day 14. The resulting patch combined wet-tissue adhesion on one surface, anti-adhesion on the other, and self-healing behavior, providing a preclinical demonstration of suture-free internal-tissue repair.

Following a similar design logic, Yu et al. [124] dissolved different concentrations of three dicarboxyl-containing vinyl monomers, namely N-acryloyl glutamic acid (AGLU), N-acryloyl aspartic acid (AASP) and N-acryloyl amidomalonic acid (AAMI), in water and prepared PAGLU, PAASP, and PAAMI hydrogels via photoinitiated free radical polymerization. Among the three formulations, the PAASP hydrogel demonstrated the strongest adhesive properties, together with high toughness, elasticity, and fatigue resistance. In vitro, PAASP remained structurally stable for 7 days in simulated gastric fluid and retained adhesion after exposure to phosphate-buffered saline or simulated gastric fluid. To impart non-adhesive functionality, the authors used a paper-based Fe^3^⁺ transfer-stamping method to create an asymmetric interface. The unmodified adhesive PAASP hydrogel reduced blood loss in mouse liver-injury models. After Fe^3^⁺ transfer stamping, the resulting Janus patch covered a 5-mm gastric incision in a rodent model, and no gross adhesion to surrounding tissue was observed at day 7. This design offers a strategy for emergency hemostasis and visceral repair.

Despite the effective sealing and anti-adhesion performance of these preformed Janus patches, their solid form can limit minimally invasive application, and a physical barrier alone does not directly address macrophage recruitment associated with adhesion formation. To address these limitations, Wu et al. [125] developed an injectable, photocurable hyaluronic acid-based asymmetric-adhesive hydrogel (HAD) functionalized with catechol and methacrylate groups. The HAD precursor was applied before curing, allowing catechol groups to interact with nucleophilic groups on the tissue surface. Subsequent UV crosslinking maintained the tissue-facing attachment while restricting catechol mobility at the outward-facing surface, which became non-adhesive. The hydrogel formed within 3–5 s under 365-nm UV irradiation in vitro, and laparoscopic delivery was demonstrated in a rabbit cecal-injury model. In addition, the authors reported that the polyanionic hyaluronic acid matrix interacted with macrophage scavenger receptor 1 and reduced the accumulation of GATA6⁺ cavity macrophages, collagen deposition, and postoperative adhesions in rat and rabbit cecal-injury models. This study provides anti-adhesion evidence but does not demonstrate closure of a gastrointestinal perforation.

Janus materials address a requirement that conventional symmetric sealants cannot readily satisfy: strong fixation at the defect and low adhesion toward adjacent tissues. Preformed patches provide well-defined opposing surfaces, whereas injectable or photocurable systems may be more suitable for minimally invasive treatment of irregular defects. However, the evidence reviewed here is limited to rodent and rabbit models. Translation will require evaluation of minimally invasive deployment, correct orientation of the adhesive surface, durable sealing under gastrointestinal motion and fluid exposure, photocuring safety where applicable, and degradation that does not compromise tissue repair.

### Inflammatory bowel disease

IBD is a group of chronic, relapsing inflammatory conditions of the bowel, comprising mainly ulcerative colitis and Crohn’s disease. UC is characterized by continuous inflammation of the colonic mucosa, usually beginning in the rectum and extending proximally, whereas CD causes discontinuous transmural inflammation that may affect any part of the GI tract [126, 127].

Current IBD treatments include aminosalicylates, corticosteroids, conventional immunomodulators, biologic agents, and targeted small-molecule therapies. Antibiotics are used for selected indications rather than as routine primary treatment. However, some patients do not respond initially, lose response over time, or experience treatment-related adverse effects. More selective local treatment and sustained mucosal repair therefore remain important clinical needs [126, 127].

#### Inflammation-targeted drug delivery

Local control of excessive intestinal inflammation is a central therapeutic objective in IBD. Monoclonal antibodies can neutralize extracellular inflammatory mediators, whereas nucleic acid therapeutics can reduce the expression of selected mediators or regulate pathways involved in inflammation and tissue repair.

Monoclonal antibodies and nucleic acid therapeutics face distinct delivery barriers. Systemically administered monoclonal antibodies may cause systemic adverse effects and induce anti-drug antibodies, whereas orally delivered antibodies are susceptible to gastric acid and enzymatic degradation. Nucleic acid therapeutics face additional barriers, including poor membrane permeability, limited in vivo stability, and inefficient cytoplasmic delivery [128, 129]. Representative studies illustrate oral and systemic delivery approaches intended to preserve biologic activity, increase intestinal exposure, or enable intracellular release.

Delivery of monoclonal antibodies

TNF-α contributes to intestinal inflammation in both UC and CD. Infliximab (IFX), a chimeric monoclonal antibody that neutralizes human TNF-α, is an established treatment for moderate-to-severe IBD. Despite their efficacy, systemically administered anti-TNF-α antibodies face clinical limitations. Anti-TNF treatment can increase susceptibility to serious infections, and anti-drug antibodies (ADAs) can lower circulating drug concentrations, increase clearance, and contribute to infusion reactions or loss of response. Immunogenicity is not caused solely by intravenous administration and is also influenced by the dosing regimen, concomitant immunomodulatory treatment, and patient-related factors. Infliximab additionally requires repeated intravenous infusions, which increase treatment burden [126–128]. Oral delivery could avoid infusions and increase local luminal exposure, although reductions in systemic exposure and immunogenicity must be demonstrated rather than assumed. However, gastric acidity and proteases can degrade orally administered antibodies, and a formulation must reach and remain at active inflammatory lesions despite intestinal clearance and variable disease distribution [129].

To address these oral delivery barriers, Li et al. [130] assembled negatively charged IFX nanocomplexes from carboxymethyl chitosan and chitosan and incorporated them into calcium alginate microparticles to form IFX-loaded nano-in-microparticles (IFX-NM). The formulation limited antibody release at pH 1.2 and released structurally intact IFX at intestinal pH. In mice with acute colitis induced by 2,4,6-trinitrobenzenesulfonic acid (TNBS), orally administered nano-in-microparticles containing cyanine 5-labeled immunoglobulin G (IgG-Cy5) produced a stronger and more persistent colonic fluorescence signal than free labeled immunoglobulin G. Because IgG-Cy5 was used in place of IFX for imaging, these results support gastrointestinal retention of the carrier but do not directly establish the pharmacokinetics or tissue distribution of IFX itself. Oral IFX-NM improved disease, histological, and barrier-related measures, with several outcomes comparable to those obtained with intraperitoneal free IFX.

In a second approach, Li et al. [131] prepared thin-shell microcapsules from alginate modified with aminoethyl methacrylate (Alg-AEMA). The double-crosslinked Alg-AEMA network was formed through Ca^2^⁺-mediated ionic crosslinking followed by UV-initiated covalent crosslinking. Because both mechanisms acted within the same modified alginate shell, the material is more accurately described as a dually crosslinked network than as two separate interpenetrating networks. The released IFX retained its measured secondary structure. More than 85% of the antibody remained encapsulated after 2 h at approximately pH 5, but this condition did not reproduce typical fasting gastric acidity. The animal protocol also included buffering of the stomach to reduce acid exposure, and direct protection against digestive proteases was not demonstrated. In a dextran sulfate sodium (DSS)-induced mouse colitis model, orally administered IFX-loaded microcapsules improved disease activity, colon length, histology, epithelial-barrier markers, and inflammatory measurements. Several measured outcomes were similar to those observed after intravenous IFX at the same dose. Sequencing of 16 S ribosomal RNA genes also identified treatment-associated changes in microbial richness and relative abundance, including increases in some Lachnospiraceae-related taxa. These associations do not establish that the microcapsules directly regulated the microbiome or that microbial changes caused the therapeutic response.

Both studies evaluated IFX in conventional mouse colitis models, without large-animal or human validation. Because IFX has been reported not to bind murine TNF-α directly, the improvements observed in these models do not by themselves establish target-specific neutralization of murine TNF-α [132]. These studies support formulation-level proof of concept but do not directly validate the intended anti-human-TNF mechanism or clinical efficacy. Future studies should use a cross-reactive surrogate antibody, a human TNF-expressing model, or another model that can establish target-specific activity.

##### Delivery of nucleic acid therapeutics

Nucleic acid therapeutics regulate gene expression through sequence-directed mechanisms and can address targets that are difficult to modulate with antibodies or small molecules. For example, small interfering RNA (siRNA) engages the RNA interference (RNAi) pathway to promote sequence-specific degradation of target messenger RNA. The duration and safety of gene regulation vary across nucleic acid modalities and delivery platforms [133, 134]. However, nucleic acid drugs, particularly siRNA, face substantial delivery barriers. Naked siRNA is hydrophilic and polyanionic and therefore crosses cell membranes inefficiently. It is also susceptible to nuclease degradation and rapid clearance. After cellular uptake, only a small fraction may escape from endosomes and reach the cytoplasm, where RNAi occurs [135, 136]. Biomaterial carriers can protect nucleic acids and alter their tissue distribution; some formulations are designed to enhance cellular uptake and endosomal escape [137]. However, the carrier can itself introduce off-target distribution, immune activation, or toxicity and must be evaluated together with the nucleic acid cargo.

Guo et al. [138] synthesized reactive oxygen species (ROS)-responsive cationic lipids containing a zinc-dipicolylamine head group, a thioketal linkage, and hydrophobic alkyl tails. The selected ZnDPA-14 formulation formed approximately 100-nm lipoplexes with TNF-α siRNA. Hydrogen peroxide cleaved the thioketal linkage and promoted siRNA release. In macrophages, the formulation was associated with lower intracellular ROS, TNF-α messenger RNA, and inflammatory cytokine levels. In an acute DSS colitis model, PEGylated lipoplexes were administered intravenously for six consecutive days. Imaging showed enrichment in intestinal tissue, but signals were also detected in the kidneys, liver, and lungs. Treatment improved disease activity, colon length, inflammatory markers, and histological findings. Hematology, serum biochemistry, and major-organ histology showed no marked abnormalities during the seven-day experiment.

For oral nucleic acid delivery, access to epithelial or subepithelial cells depends on particle size, surface chemistry, mucus interactions, disease-related changes in barrier permeability, and the intended cellular target. A particle size below 100 nm may facilitate transport in some experimental systems, but it is not a universal requirement that guarantees mucus or tissue penetration [28]. To address successive GI barriers, Li et al. [139] used microRNA-320 (miR-320) as a model cargo to prepare multistage-responsive nanocomplexes. MiR-320 was first enclosed in polymeric nanocapsules formed using a disulfide-containing crosslinker. The positively charged nanocapsules were then assembled with alginate and compacted through Ca^2^⁺ crosslinking. The complete alginate complexes measured approximately 300–900 nm, whereas the nanocapsules released from them measured 24–36 nm. Enzyme- and redox-responsive structural changes were demonstrated in vitro using trypsin and mercaptoethanol, respectively. After oral administration to mice, carrier-associated fluorescence was concentrated in the colon within 2 h and extended into the submucosal and muscular layers. In the acute DSS model, dosing began 48 h before DSS exposure and continued during disease induction, representing a preventive or early-intervention design. Treatment improved disease, inflammatory, and histological measures and was accompanied by lower myeloperoxidase activity and reduced phosphorylation of IκBα and AKT. Major-organ histology at the end of the seven-day experiment showed no obvious abnormalities.

These studies represent two different delivery settings. ZnDPA-14 was administered systemically and showed intestinal enrichment together with distribution to other organs, whereas the miR-320 formulation was administered orally and used sequential structural changes to support colonic delivery. Both studies were limited to short-term acute mouse models. Further evaluation should address treatment after established disease, cell-specific delivery, durability of gene regulation, off-target effects, and repeated-dose safety.

#### Enhancement of the mucosal protective barrier

Disruption of the intestinal barrier can allow microorganisms and other luminal antigens to enter the bowel wall and contribute to dysregulated immune activation [140]. Increased intestinal permeability has also been prospectively associated with the subsequent development of Crohn’s disease [141]. Locally retained materials may protect damaged mucosa, support epithelial repair, and limit systemic exposure. Alterations in microbial communities are also associated with IBD, although their composition and contribution vary [142].

Mao et al. [143] developed a mucosa-adherent coating based on o-nitrobenzaldehyde (NB)-modified gelatin (GelNB). Upon UV light activation, photogenerated aldehyde groups reacted with amino groups (-NH_2_) on intestinal tissue surfaces, forming a thin biophysical barrier. Fluorescence imaging, repeated-washing experiments, and microscopy showed that GelNB formed a stable coating on the intestinal surface, while permeability experiments supported its barrier function against selected intestinal metabolites. In DSS- and TNBS-induced mouse models, rectally administered GelNB improved disease activity, colon length, histological findings, inflammatory markers, and tight-junction-protein expression; several measured outcomes were more favorable than those obtained with mesalazine. Furthermore, 16 S rRNA sequencing analysis of gut microbiota demonstrated that GelNB treatment restored microbial community structure across multiple taxonomic levels (class, order, family, and species), achieving a composition closely resembling that of healthy controls. GelNB therefore provided a physical barrier and was associated with lower inflammation, improved barrier markers, and altered microbial composition in the mouse models.

Some conventional hydrogel systems for IBD may be limited by insufficient targeting and low network density. These limitations may reduce local delivery and allow harmful substances to pass through gaps in the hydrogel layer, while the loaded drugs may also cause adverse effects [144]. To address these limitations, Chen et al. [145] crosslinked PVA and alginate (volume ratio 5:1) with CaCl_2_ to form highly negatively charged gel microspheres (PALMs; ζ-potential ≈ − 41.6 mV in artificial colonic fluid). The negative surface charge was intended to promote preferential adhesion to positively charged inflamed mucosa. Subsequently, they introduced cyclodextrin polymer (CPC) to develop an inflammation-targeting trigger-repair hydrogel (ITTH hydrogel). This system utilizes dynamic borate bonds to trigger in situ secondary crosslinking of PALMs, forming a dense network barrier. In an ex vivo mouse-colon flow model, the fluorescence retained on inflamed tissue after 0.5 h was approximately 70-fold higher than that on healthy tissue. In human UC biopsy samples, PALM-associated fluorescence after 2 h was more than twofold higher at inflamed than at normal sites. The in situ-formed gel also remained intact during colonic peristalsis in DSS-treated mice. CPC-triggered crosslinking began within 5 s and was complete by 13 s, producing a continuous lamellar network that reduced the passage of RhoB, BSA, *E. coli*, and *S. aureus* in vitro. In the DSS-induced colitis model, the ITTH hydrogel reduced colon shortening and splenomegaly and increased PAS-positive goblet cells. The authors reported overall therapeutic effects comparable to daily 5-aminosalicylic acid (5-ASA) and greater than those of PALMs or CPC alone, although ITTH was administered every other day.

Many experimental biomaterial strategies for IBD have been designed to address individual features of the inflamed gut, such as oxidative stress or microbiota dysbiosis. Because these abnormalities can occur concurrently, materials that act through more than one mechanism have also been explored. Xu et al. [146] developed an injectable thermosensitive and inflammation-targeting hydrogel system (TMNR). TMNR comprised a thermosensitive hydrogel (FCH) formed from HA, α-cyclodextrin (α-CD), and Poloxamer F127 and loaded with a melanin–gallium metabolic nanoregulator (MNR) prepared by chelating Ga^3^⁺ to PEGylated melanin nanoparticles (MNPs). In vitro, MNR scavenged several reactive oxygen species, while Ga^3^⁺ interfered with bacterial iron metabolism; the FCH matrix also reduced bacterial penetration. In DSS-treated mice, TMNR reduced colon shortening and disease activity, lowered TNF-α and IL-1β relative to PBS controls, and increased goblet-cell numbers. 16 S rRNA gene sequencing also showed treatment-associated changes in fecal microbial composition. These results associate the formulation with antioxidant, microbiota-related, and barrier-protective effects in this model.

Zhang et al. [147] incorporated two-dimensional titanium carbide (Ti_3_C_2_) MXene nanosheets into a carboxylated chitosan (CCS) and sodium carboxymethyl cellulose (CMC-Na) formulation without an added crosslinking agent. Acidic conditions strengthened ionic interactions between the polysaccharides and induced gel formation, whereas neutral or alkaline conditions weakened these interactions. Release was faster in simulated colonic fluid, but the contribution of specific microbiota-derived enzymes was not identified. After oral administration to colitic mice, fluorescence persisted in the colon for up to 12 h, and measurements of titanium at 12 h showed greater content in the colon than in major organs. In acute and chronic DSS models and an acute TNBS model, treatment improved clinical and histological measures. Several outcomes in the high-dose group were better than those obtained with 5-ASA in the same mouse experiments, but this does not establish clinical superiority. Treatment was also associated with increased tight-junction-protein expression, reduced markers related to NOD-like receptor family pyrin domain-containing 3 (NLRP3)/caspase − 1/gasdermin D-mediated pyroptosis, and changes in fecal microbial composition. These associations do not establish that either the signaling changes or microbiome changes caused the therapeutic response. A separate 50-day repeated-dose study showed no marked abnormalities in the reported blood measurements or major-organ histology.

To couple disease sensing with sustained mucosal repair, Ge et al. [148] engineered nonpathogenic *Escherichia coli (E. coli)* with a blood-inducible genetic circuit that produced a therapeutic living glue (TL-glue) in response to gastrointestinal bleeding. The engineered bacteria secreted the barnacle-derived adhesive protein CP43K together with the mucosal repair factor trefoil factor 3 (TFF3). CP43K formed an adhesive matrix that prolonged bacterial attachment to inflamed tissue, while TFF3 supported epithelial repair and barrier restoration. For oral delivery, the bacteria were coated with the pH-responsive enteric polymer Eudragit L100-55, which protected them during gastric transit and released them in the intestine. Bleeding-induced adhesion maintained the engineered bacteria for up to 10 days after rectal administration and 7 days after oral administration. In dextran sulfate sodium-induced and interleukin-10-knockout colitis models, TL-glue improved weight recovery, limited colonic shortening and intestinal bleeding, reduced inflammation, and restored mucosal and tight-junction integrity. Unlike static coatings or hydrogel barriers, this living material generated its adhesive matrix and therapeutic protein locally in response to a disease-associated signal.

Biomaterial applications in IBD broadly serve two therapeutic objectives: molecularly targeted delivery and restoration of the mucosal barrier. Targeted delivery systems protect antibodies, nucleic acids, and other therapeutic agents while promoting their accumulation or intracellular release at inflamed sites. Barrier-forming coatings and hydrogels instead shield the damaged epithelium and support mucosal repair, although some materials combine both functions. Engineered living systems add a further option by coupling disease sensing to local production of adhesive and therapeutic proteins. Material-system selection therefore depends on whether the primary therapeutic objective is molecular intervention, epithelial protection, or both. The evidence for the systems discussed here remains predominantly preclinical and is based mainly on short-term mouse models. The ITTH study included human tissue only for ex vivo retention testing. Variable disease distribution, microbiome-dependent cues, repeated-dose safety, and route-specific procedures remain important translational considerations.

### Gastrointestinal neoplasms

This section focuses mainly on gastric cancer and CRC, which are among the most common and lethal gastrointestinal malignancies worldwide and impose a substantial disease burden in China [149–151]. Surgery, chemotherapy, and radiotherapy remain central to treatment. Their effectiveness can be limited by treatment-related toxicity, recurrence or metastasis, and an immunosuppressive tumor microenvironment (TME) [16, 152].

Biomedical materials are being investigated to improve local drug residence, alter the distribution of systemically administered therapies, support photothermal treatment, and modulate antitumor immune responses. Their value depends on the administration route, tumor model, and ability to improve efficacy or safety relative to clinically relevant comparators.

#### Tumor-targeted drug delivery systems

The therapeutic efficacy of conventional systemic chemotherapy for gastrointestinal malignancies can be limited by poor tumor selectivity and off-target toxicity, as well as tumor-associated barriers to vascular extravasation, interstitial transport, and cellular uptake [16, 153]. Material systems developed to address these limitations use three complementary strategies: (1) localized sustained release for postoperative or accessible lesions; (2) nanoscale carriers with responsive or ligand-modified surfaces to alter tumor distribution and cellular uptake; and (3) biomimetic coatings intended to reduce immune clearance or promote tumor-cell recognition. These strategies illustrate how material selection changes with the treatment setting and administration route.

Local sustained-release systems have been investigated to prolong intraperitoneal drug retention after gastric-cancer surgery. Chen et al. [154] engineered a biodegradable, temperature-responsive poly(D,L-lactide)–poly(ethylene glycol)–poly(D,L-lactide) (PDLLA–PEG–PDLLA; PLEL) hydrogel for the co-delivery of 5-fluorouracil (5-FU) and cisplatin. Its sol-to-gel transition allowed the formulation to be injected as a liquid and to gel at physiological temperature, creating a local drug depot. In mouse models of postoperative gastric-cancer recurrence and peritoneal metastasis, the co-loaded hydrogel reduced tumor burden and produced fewer systemic-toxicity findings than the free-drug regimen under the study conditions.

Wu et al. [155] engineered an injectable, dynamically crosslinked hydrogel (PFA/PPLL) for the co-delivery of metformin and 5-FU. Aldehyde-functionalized four-arm poly(ethylene glycol) (PFA) formed dynamic Schiff-base bonds with amino groups on poly(ethylene glycol)-poly(L-lysine) (PPLL), allowing rapid in situ gelation after the two precursor solutions were mixed. In vitro, the hydrogel degraded and released both drugs faster at pH 6.0 than at pH 7.4, consistent with the acid sensitivity of the Schiff-base crosslinks. In BALB/c mice bearing subcutaneous C26 tumors, a single peritumoral injection of the co-loaded hydrogel produced a reported tumor-inhibition ratio of 95.1% after 21 days. Major-organ histology showed less liver and kidney injury than the free metformin/5-FU combination.

Local hydrogels are mainly applicable to postoperative or accessible lesions, whereas unresectable or metastatic disease may require systemic delivery. Xiao et al. [156] prepared a carrier-free nanosuspension (SICN) by co-assembling irinotecan hydrochloride and curcumin with Poloxamer 105. The particles were negatively charged at physiological pH and became positively charged under acidic conditions, a change intended to reduce macrophage uptake during circulation and increase tumor-cell uptake after entry into the tumor. In the HT-29 xenograft mouse model, SICN altered irinotecan biodistribution, showing lower relative accumulation in the stomach and intestine than free irinotecan; severe diarrhea was not observed in the SICN-treated group.

Some nanocarriers have also been designed to combine drug delivery with modulation of the tumor immune microenvironment. Although FOLFOX (folinic acid, 5-FU, and oxaliplatin) is a standard clinical regimen for CRC, its use is limited by incomplete responses [151], treatment-related toxicity [157], and nonspecific targeting [158]. Guo et al. [159] used aminoethyl anisamide-modified polyethylene glycol (PEG)-lipid nanoparticles to deliver active components of the folinic acid, 5-FU, and oxaliplatin regimen. Nano-Folox contained a precipitated complex formed from an active oxaliplatin derivative and folinic acid. In an orthotopic CRC mouse model, Nano-Folox prolonged the circulation of the platinum component and increased its tumor accumulation; when combined with free 5-FU, it produced stronger antitumor and immune responses than the tested conventional FOLFOX regimen. Addition of programmed death ligand 1 (PD-L1) blockade further reduced liver metastases in a separate experimental CRC liver-metastasis model [159]. The group subsequently developed Nano-FdUMP to deliver 5-fluoro-2′-deoxyuridine − 5′-monophosphate, an active metabolite of 5-FU [160]. Nano-FdUMP increased reactive oxygen species (ROS), and its combination with Nano-Folox increased markers of oxaliplatin-associated immunogenic cell death (ICD) and inhibited tumor growth in orthotopic CRC and hepatocellular carcinoma models. Combination with anti-PD-L1 treatment also prolonged survival in a mouse model of CRC liver metastasis. These studies provide preclinical evidence for nanoparticle-assisted chemo-immunotherapy but do not establish that the formulations are safer or more effective than FOLFOX in patients.

Despite advances in PEGylation and surface functionalization, synthetic carriers remain susceptible to clearance by the mononuclear phagocyte system. Cell membrane coatings have been investigated as a means of reducing phagocytic clearance. Zhang et al. [51] prepared a cancer-cell-membrane-coated nanoplatform (M@NPs/miR365). miR365 mimics were electrostatically complexed with ε-poly-L-lysine-functionalized poly(citrate-peptide) cores, which were then coated with membranes from MC38 cells. The coating was intended to reduce macrophage uptake through CD47 and increase interactions with homologous tumor cells. Following intravenous administration to mice bearing subcutaneous MC38 tumors, the membrane-coated formulation showed greater tumor-associated fluorescence and stronger tumor-growth inhibition than the tested uncoated formulations.

To extend oral nanomedicine from conventional drug delivery to in situ gene editing, Zhao et al. [161] developed an oral CRISPR-Cas9 delivery system targeting tumor necrosis factor receptor-associated protein 1 (TRAP1), a mitochondrial chaperone associated with chemoresistance in CRC. Hyaluronic acid (HA) was conjugated with trimethylamine N-oxide (TMAO) and used to coat a poly(β-amino ester) (PBAE)-plasmid nanocomplex, producing the HA–TMAO-modified PBAE-based DNA nanocomplex (HTPBD). The hydrated zwitterionic coating shielded the positive charge of the gene carrier, reduced nonspecific interactions with mucus, and protected the plasmid during gastrointestinal transit. HTPBD subsequently crossed the intestinal epithelium through organic cation/carnitine transporter 2-mediated transcytosis and accumulated in CRC tissue. CRISPR-Cas9-mediated TRAP1 disruption promoted cyclophilin D translocation and sustained opening of the mitochondrial permeability transition pore, thereby sensitizing tumor cells to 5-FU-induced necrosis and immune activation. In an orthotopic CRC model, TRAP1-targeting HTPBD combined with 5-FU achieved a tumor inhibition rate of 93.3%, compared with 35.8% for 5-FU alone. When further combined with anti-programmed cell death protein 1 (PD-1) treatment in a chemoresistant orthotopic model, the system prolonged median survival from 17 to 44 days. It also reduced intestinal tumor burden in *Apc*^Min/+^ mice with spontaneous tumors.

Curcumin-based materials have also been examined as radiosensitizers. Xu et al. [162] prepared self-assembled curcumin nanofibers that increased intratumoral curcumin retention and enhanced the response to ionizing radiation in colorectal tumor models.

These studies cover distinct clinical settings. Local hydrogels may be useful for resection beds, peritoneal disease, or directly accessible tumors, whereas systemically administered nanoparticles are intended to alter circulation, tumor accumulation, or cellular uptake. Oral systems must additionally withstand GI conditions and cross mucus and epithelial barriers. Comparisons across these approaches should therefore consider the administration route, tumor location, disease stage, and relevant clinical comparator rather than particle accumulation alone.

#### Photothermal therapy

Conventional systemic chemotherapy for CRC can be limited by inadequate tumor exposure and systemic toxicity. To improve treatment efficacy, multimodal strategies combining chemotherapy with radiotherapy [163], gene therapy [164], photothermal therapy (PTT) [165], or immunotherapy [166] have been investigated. PTT converts absorbed light into heat and can also be combined with photodynamic therapy, catalytic therapy, or immune modulation [167]. Although PTT offers a degree of spatial and temporal control, its performance depends on both the accumulation of photothermal agents within the tumor and the effective delivery of light to the target tissue [168]. Insufficient tumor accumulation [71], suboptimal heat generation [167], limited tissue penetration of incident light, heterogeneous agent distribution, heat diffusion, and the persistence of some inorganic components may restrict therapeutic efficacy and increase off-target injury. Biomaterial carriers are being developed to improve localized photothermal-agent delivery and support combination therapy.

Macroscopic in situ hydrogels offer one approach to retaining photothermal agents locally. For instance, Zheng et al. [169] engineered an injectable, temperature-sensitive chitosan/MBP/DOX (CMD) hydrogel for combined photothermal and chemotherapeutic treatment of colon cancer. The hydrogel contained MoS_2_/Bi_2_S_3_-PEG (MBP) nanosheets as photothermal agents and doxorubicin (DOX) within a chitosan/β-glycerophosphate (β-GP) matrix. This matrix remained flowable at room temperature and gelled at 37 °C. DOX was gradually released from the CMD hydrogel, with the release profile influenced by both pH and temperature. Under NIR-I and NIR-II irradiation, the MBP nanosheets generated local heating, allowing the system to combine photothermal treatment with sustained local DOX delivery. The CMD hydrogel remained solid after heating at 80°, whereas the agar- and alginate-based comparator hydrogels became fluid. This resistance to heat-induced melting may help maintain the local hydrogel depot during photothermal treatment. The chitosan-based hydrogel also inhibited *E. coli* growth in vitro.

Although macroscopic hydrogels can retain photothermal agents at the tumor site, conventional PTT often requires temperatures above 50 °C for tumor ablation, which can damage surrounding tissues and provoke inflammation that may promote tumor progression [170, 171]. Mild PTT (approximately 45 °C) [172, 173] can instead enhance intratumoral drug penetration and cellular uptake and sensitize tumor cells to chemotherapy [174, 175], while chemotherapy compensates for the limited tumor-killing effect of low-temperature heating [176]. Based on this rationale, Chen et al. [177] developed a supramolecular drug-loaded nanocomposite hydrogel (NCH). Oxaliplatin (OxPt) was encapsulated within the cavity of cucurbit[7]uril (CB[7]) to form a supramolecular complex (OxPt@CB[7]), which reduced OxPt cytotoxicity toward human umbilical vein endothelial cells in vitro, while spermine displaced OxPt from the complex. This complex was combined with gold nanoparticles (AuNPs) as photothermal agents and a self-healing chitosan/oxidized konjac glucomannan (CS/OKGM) hydrogel, rendering the system injectable. After intratumoral injection in mice bearing subcutaneous HCT116 tumors, mild photothermal treatment at approximately 45 °C combined with OxPt@CB[7]-NCH limited tumor growth more than either component treatment. In a separate incomplete-resection model using the same subcutaneous tumor system, local OxPt@CB[7]-NCH reduced tumor recurrence. The CS/OKGM matrix also reduced postoperative adhesion in a separate rat abdominal wall–cecum defect model. Body weight, major-organ histology, and blood analyses showed no marked abnormalities after the combination treatment.

Nanoscale platforms have also been developed to combine PTT with complementary modalities, including photodynamic therapy (PDT) and nanozyme-mediated catalytic therapy [178, 179]. Zhang et al. [180] engineered multimodal nanoprobes (OMCAPs@rBSA-FA@IR780) integrating photothermal, photodynamic, and catalytic therapies. Mesoporous carbon nanospheres (MCNs) were doped with small AuNPs to form mesoporous carbon–gold hybrids (MCAPs), which were then acid-oxidized and carboxylated to generate carboxylated mesoporous carbon–gold hybrids (OMCAPs). The OMCAPs were surface-modified with reduced bovine serum albumin (rBSA) conjugated with folic acid (FA) and then loaded with IR780 to obtain OMCAPs@rBSA-FA@IR780. MCNs were selected because of their large pore volume, favorable biocompatibility, electrical conductivity, and broad UV–visible–NIR absorption. In the resulting nanoplatform, OMCAPs provided a large surface area and abundant carboxyl groups for IR780 loading, the incorporated AuNPs acted as nanozymes that catalyzed H_2_O_2_ to generate hydroxyl radicals (•OH), and FA provided gastric-tumor-targeting functionality. IR780 further contributed to NIR fluorescence imaging, PDT, and PTT under 808 nm laser irradiation. In MGC-803 tumor-bearing mice, the nanoprobes supported tumor-targeted imaging and multimodal therapy.

PTT may also induce immunogenic cell death (ICD) and promote antitumor immune responses [181, 182]. However, programmed death-ligand 1 (PD-L1)-mediated T-cell inhibition can limit these responses, providing a rationale for combining PTT with modulation of immune checkpoints [183]. In this context, Wan et al. [184] developed a multifunctional biomimetic nanoplatform, Fe_3_O_4_@PDA@CaCO_3_-ICG@CM. The core–shell particles combined magnetic Fe_3_O_4_, polydopamine (PDA), acid-responsive CaCO_3_, and indocyanine green (ICG) and were coated with engineered EL4 cell membranes containing lymphocyte function-associated antigen 1 (LFA-1), PD-1, transforming growth factor-β receptor, and Fas ligand. After tail-vein administration in mice bearing bilateral subcutaneous CT26 tumors, Fe_3_O_4_ provided magnetic guidance, whereas interactions between LFA-1 and intercellular adhesion molecule 1 and PD-1/PD-L1 interactions contributed to tumor association. Fluorescence imaging showed the highest tumor-associated signal when the membrane-coated particles were combined with an external magnet. Fas ligand-mediated tumor-cell killing was associated with activation of the caspase − 8 pathway. In magnet-treated mice, PDA and ICG increased the temperature of CT26 tumors to approximately 52.5 °C after 5 min of 808-nm irradiation. Treatment increased calreticulin exposure and high-mobility group box 1 release and was associated with dendritic cell (DC) maturation, greater intratumoral CD4⁺ and CD8⁺ T-cell abundance, reduced regulatory T-cell infiltration, and increased granzyme B. In the bilateral CT26 model, the formulation combined with magnetic guidance and laser irradiation inhibited both the irradiated primary tumor and the nonirradiated distant tumor. The treatment also increased the abundance of CD44⁺CD62L⁺ central memory T cells. Following intravenous rechallenge with CT26 cells, treated mice showed markedly reduced pulmonary nodule formation, and histological examination revealed no obvious lung metastases, consistent with an immune-memory response in this mouse model.

These studies show that thermosensitive depots, nanocomposite hydrogels, carbon-gold nanoprobes, and biomimetic particles can combine heat generation with chemotherapy, catalytic activity, or immune modulation. Local hydrogels are most relevant to accessible tumors and surgical settings. Systemic nanoscale formulations may reach less accessible lesions but introduce additional barriers involving biodistribution, tumor penetration, manufacturing, and clearance. Long-term evaluation is particularly important for formulations containing gold, iron oxide, carbon, or other persistent inorganic components.

#### Cancer immunotherapy

Cancer immunotherapy aims to mobilize antitumor immune responses and can produce durable clinical benefit in selected patients. However, many gastrointestinal tumors, particularly immune-cold tumors, respond poorly because of low tumor immunogenicity, limited immune-cell infiltration, and immunosuppressive signaling within the TME [185]. In CRC, the clinical activity of immune checkpoint inhibitors differs markedly according to molecular subtype and is substantially greater in mismatch repair-deficient (dMMR) or microsatellite instability-high (MSI-H) tumors than in most mismatch repair-proficient (pMMR) or microsatellite-stable (MSS) tumors [186, 187]. Biomaterial-assisted strategies are being investigated to induce immunogenic cell death (ICD), deliver tumor antigens or nucleic acids, activate innate immune sensing, and modulate immune checkpoints [188, 189]. Pattern recognition receptors (PRRs) detect pathogen-associated or damage-associated molecular patterns and initiate innate immune signaling [190]. Among them, cyclic GMP-AMP synthase (cGAS) senses cytosolic microbial or host-derived DNA and activates the stimulator of interferon genes (STING) pathway [191].

Some chemotherapeutic agents induce DNA damage that can activate cGAS–STING signaling, but poor solubility and off-target toxicity can limit their use. To address these limitations, Cao et al. [192] engineered a ROS-responsive nanoplatform to deliver a highly hydrophobic camptothecin–platinum(IV) prodrug (CPT-Pt(IV)). The formulation was designed to increase tumor-localized DNA damage and activate cGAS–STING signaling. In a subcutaneous CT26 colorectal tumor model, the nanoplatform activated the cGAS-STING pathway, promoted DC maturation and intratumoral CD8⁺ T-cell infiltration, and showed low systemic toxicity at the tested dose.

A complementary approach is to combine an ICD-inducing chemotherapeutic agent with a carrier that provides an additional immunostimulatory mechanism. Mitoxantrone (MIT) can induce ICD, although the nonspecific distribution of free MIT may expose normal tissues and immune cells to cytotoxic effects [193]. To improve tumor-localized MIT delivery and amplify ICD, Li et al. [194] developed mitoxantrone-encapsulated ZIF-8 (MIT@ZIF-8) through a one-pot aqueous coordination process involving Zn^2^⁺ and 2-methylimidazole (2-MIm). ZIF-8 served both as a pH-responsive carrier for mitoxantrone (MIT) and as a source of immunomodulatory Zn^2^⁺. In the acidic TME, disruption of the Zn^2^⁺-imidazole coordination bonds led to the release of MIT and Zn^2^⁺. MIT exerted its chemotherapeutic effect, whereas Zn^2^⁺ induced caspase − 1/gasdermin D (GSDMD)-dependent pyroptosis. Their combined actions increased calreticulin exposure and high-mobility group box 1 release, thereby amplifying immunogenic cell death. In vivo, MIT@ZIF-8 inhibited the growth of immunologically “hot” CT26 colon tumors and “cold” RM-1 prostate tumors, increased CD8⁺ T-cell infiltration, reduced the proportion of regulatory T cells, and preserved immune-cell viability in tumor-draining lymph nodes. The investigators further evaluated MIT@ZIF-8 in combination with anti-cytotoxic T-lymphocyte-associated protein 4 (CTLA-4) therapy in the RM-1 model. Whereas anti-CTLA-4 treatment alone failed to inhibit tumor progression, the combination significantly suppressed tumor growth and prolonged survival. In the tested models, MIT@ZIF-8 combined MIT delivery with Zn^2^⁺-associated pyroptotic signaling and increased the response of RM-1 tumors to anti-CTLA-4 therapy.

Material-assisted tumor vaccination provides another approach to stimulating antitumor immunity. To overcome the weak immunogenicity of traditional autologous vaccines, Ke et al. [195] developed a bifunctional fusion membrane hydrogel. They constructed fusion membrane nanoparticles (FM-NPs) by integrating autologous tumor cell membranes with membrane extracts derived from *Mycobacterium phlei*, which provided bacterial adjuvant components. These nanoparticles were then co-encapsulated with granulocyte–macrophage colony-stimulating factor (GM-CSF) within a sodium alginate (ALG) matrix to form an in situ DC vaccine. Following subcutaneous injection, the sodium alginate hydrogel was designed to release GM-CSF rapidly and recruit immature DCs while retaining the bacterial components intended to promote DC maturation and tumor-antigen loading. This localized depot was intended to limit systemic exposure to the bacterial components. In a mouse mammary tumor model, the system promoted DC maturation and improved survival when combined with PD-1 blockade; activity was also observed in gastric cancer organoids. Thus, this study provides platform-level support for a material-assisted cancer vaccine, but its therapeutic efficacy requires direct validation in orthotopic GI tumor models.

Checkpoint modulation has also been investigated. Inhibition of proprotein convertase subtilisin/kexin type 9 (PCSK9) has been reported to enhance responses to anti-PD-1 therapy [196, 197]. Delivery carriers can protect aptamers and small interfering RNAs and improve their delivery to tumors [134]. Guo et al. [198] engineered a FA-modified tetrahedral DNA nanoplatform (TDN-FA/PL1/Pcsk9 siRNA). FA was conjugated to the constituent DNA strands through a hydroxyprolinol-based linker to promote uptake by folate receptor-positive CRC cells. The tetrahedral DNA served as a carrier for the PD-L1-binding aptamer PL1 and Pcsk9 siRNA, which reduced Pcsk9 expression. In a subcutaneous CT26 mouse model, the formulation was administered intravenously every 2 d for 10 doses. At 1 h after injection, FA-modified TDNs produced greater tumor-associated fluorescence than non-FA-modified TDNs. The combined formulation inhibited tumor growth by 83.48% and was associated with lower PCSK9 expression and higher major histocompatibility complex class I (MHC-I), interferon-γ (IFN-γ), and granzyme B levels in tumors. Body weight, serum liver and kidney markers, and major-organ histology showed no marked differences from the control group during the study.

Modulating the gut microbiome has also been investigated in colorectal cancer [199]. Some probiotics produce metabolites with reported antitumor effects, but gastric and intestinal conditions can reduce their viability during oral delivery. To overcome this, Wang et al. [200] encapsulated *Lactobacillus reuteri* in a gelatin–inulin hydrogel to form Lr@GI. In simulated gastric fluid, encapsulation increased bacterial viability 1.6-fold after 2 h, and the capsule released the bacteria under intestinal conditions. In an orthotopic colon-tumor model, Lr@GI inhibited tumor growth and increased the response to 5-FU. Treatment was associated with glutathione depletion, increased ROS, NLRP3 activation, M1-like macrophage polarization, higher zonula occludens-1 (ZO-1) expression, and changes in short-chain-fatty-acid-producing taxa and metabolites. No marked systemic-toxicity findings were reported at the assessed endpoints.

Materials used in gastrointestinal tumors share several requirements with those used in nonmalignant GI disease, including protection from physiological conditions, local residence, controlled release, and reduced off-target exposure. Their principal therapeutic goals differ, however. Materials for ulcers, perforations, and IBD primarily protect, seal, or repair tissue, whereas tumor-directed systems need to selectively eliminate malignant cells and may also need to overcome vascular, interstitial, cellular, and immunological barriers. Local hydrogels are most applicable to resection beds, peritoneal disease, or accessible lesions, whereas systemic, oral, and biomimetic nanoparticles address different distribution barriers at the cost of greater formulation and safety complexity. Most evidence remains based on transplantable mouse tumors, including many subcutaneous models that do not reproduce the anatomy, microbiome, metastatic pattern, or immune environment of human gastrointestinal cancer [201]. Future studies should use clinically relevant orthotopic or spontaneous models and comparators and directly assess tumor exposure, long-term material fate, and systemic toxicity.

### Obesity and gut-associated metabolic disorders

Obesity is a chronic systemic metabolic disease that impairs health and shortens life expectancy [202–204]. Although it is not generally classified as a gastrointestinal (GI) disease, the GI tract contributes to its pathophysiology and provides an important interface for biomaterial-based intervention. Obesity has multiple causes, and excessive energy intake is an important contributor [202]. Through nutrient digestion and absorption, interactions between the resident microbiota and host metabolism, and gut-associated inflammatory signaling, the GI tract can influence energy homeostasis and metabolic dysfunction. Biomaterials can act locally within the intestinal lumen or at the mucosal surface, respond to GI conditions, or facilitate the oral delivery of metabolic therapeutics. This section focuses on obesity and related metabolic disorders for which the underlying mechanism or biomaterial intervention is connected to the GI tract.

#### Modulation of the gut microbiota and intestinal microecology

As the primary site of nutrient digestion and absorption, the gut contributes to host energy balance through nutrient processing and interactions with the resident microbiota. Early studies associated obesity with altered proportions of *Firmicutes* and *Bacteroidetes* and showed that an obesity-associated microbiota could increase energy harvest and fat accumulation in germ-free mice [205, 206]. Later reanalyses of human datasets did not find a reproducible association between obesity and the relative abundance ratio of *Firmicutes* and *Bacteroidetes*. This ratio should therefore not be interpreted as a general marker of obesity [207]. Short-chain fatty acids (SCFAs) participate in intestinal signaling and host metabolism, but their relationship with obesity depends on the individual metabolite, its site of production and absorption, and the host metabolic context [4, 208]. The links among intestinal microorganisms, microbial metabolites, and host metabolism have led to approaches that deliver compounds to the colon or modify the survival and mucosal behavior of therapeutic bacteria.

Two studies applied related chitosan/pectin systems to increase the colonic exposure of small molecules intended to interact with the intestinal microbiota. Guo et al. [209] used berberine-loaded nanoparticles in high-fat diet (HFD)-fed male hamsters, whereas Lu et al. [210] used a related formulation to deliver poorly soluble nitidine chloride in male mice with HFD-induced NAFLD-like changes. Both formulations limited drug release during sequential in vitro gastric and small-intestinal exposure and released more drug after transfer to a pH 7.4 medium containing cecal filtrate. Five weeks of treatment with the berberine formulation reduced adiposity and improved metabolic, hepatic, and intestinal barrier measurements, whereas four weeks of treatment with the nitidine chloride formulation improved serum lipid and aminotransferase levels and attenuated hepatic and colonic injury. Both studies also reported changes in bacterial composition. However, pH, exposure time, and microbial content changed simultaneously in the sequential release experiments, and neither study established that the microbial changes caused the metabolic improvements.

Rather than delivering small molecules to the colon, therapeutic bacteria can be surface-modified to control their interaction with intestinal mucus. Chen et al. [211] used polydopamine to attach 2 kDa amino-terminated poly(ethylene glycol) (PEG) to live *E. coli* Nissle 1917 and *Clostridium butyricum (C. butyricum)*. PEG coating reduced mucin binding and increased bacterial movement through reconstituted mucus. The coating also increased intestinal bacterial retention measured 1 h after oral gavage in mice. In HFD-fed prediabetic mice, PEGylated *C. butyricum* increased colonic goblet-cell numbers and increased the expression of the tight-junction proteins occludin and zonula occludens 1 relative to untreated HFD controls. However, PEGylated *C. butyricum* alone did not lower fasting blood glucose. Improvements in fasting glucose, glucose and insulin tolerance, adipose tissue measurements, and hepatic lipid accumulation were observed with a combined formulation containing both PEGylated *C. butyricum* and taurodeoxycholate. The findings support mucus-directed bacterial delivery and the activity of the combined formulation.

Improving bacterial tolerance to gastrointestinal and oxidative conditions provides another approach. Mao et al. [212] combined bacterial adaptation with a protective surface coating. An *E. coli* Nissle 1917 variant was selected through repeated exposure to sublethal hydrogen peroxide and subsequently coated with fructooligosaccharide and calcium carbonate. The resulting REcN-F/Ca formulation retained greater viability after sequential exposure to simulated gastric, intestinal, and oxidative colonic media. Male mice received REcN-F/Ca throughout ten weeks of HFD feeding. Relative to untreated HFD controls, REcN-F/Ca reduced weight gain, epididymal fat mass, homeostatic model assessment of insulin resistance, histological hepatic steatosis, and adipose inflammatory measurements and improved glucose handling. Fecal analyses showed partial restoration of microbial richness and diversity, enrichment of *Blautia* and *Lachnospiraceae*, and higher concentrations of several SCFAs. Pharmacological inhibition of peroxisome proliferator-activated receptor-α (PPARα) reduced several metabolic benefits, supporting the involvement of PPARα signaling.

Evidence for biomaterial-based microbiota interventions remains predominantly preclinical. Future studies should distinguish preventive effects from the treatment of established metabolic disease, directly test the contributions of microbiota and microbial metabolites, and examine the durability and safety of repeated administration. Engineered living products also require assessment of genetic stability, antimicrobial resistance, shedding, and biocontainment. Because obesity is often accompanied by impaired glucose homeostasis, GI-mediated interventions may also involve local regulation of carbohydrate digestion and oral delivery of therapeutics intended to achieve systemic glucose control.

#### Glycemic regulation and diabetes management

GI-mediated glycemic regulation can be pursued through oral systems that protect peptide therapeutics and support their intestinal release and absorption, or through formulations that locally regulate carbohydrate digestion or microbiota-related processes.

Oral delivery of peptide drugs is limited by degradation in gastric and intestinal fluids, restricted transport through mucus and low epithelial permeability [8, 22, 213]. To address these issues, various nanocarrier- and hydrogel-based strategies have been developed. For example, Zhou et al. [47] developed a multicomponent system for oral exendin-4 delivery. The system used NH_2_-MIL-101(Fe) metal–organic framework (MOF) nanoparticles to load exendin-4, applied a zwitterionic hydrogel coating, and enclosed the particles in Eudragit L100-55-coated enteric capsules containing sodium bicarbonate and citric acid. The mesoporous structure of the MOF enabled an exendin-4 loading capacity of 0.402 ± 0.005 g/g. Mucin-based diffusion assays and Caco-2 and HT29-MTX-E12 cell models indicated that the zwitterionic coating reduced interactions with mucin, while the increased hydrophobicity of the N-isopropylacrylamide-containing coating at 37 °C promoted cellular uptake. The Eudragit coating was designed to dissolve above pH 5.5, and the capsules disintegrated within approximately 3 min at pH 7.4 as carbon dioxide was generated. In a diabetic rat model, a single oral dose maintained increased plasma exendin-4 concentrations for more than 8 h, increased endogenous insulin secretion, and lowered blood glucose, with a relative pharmacological availability of 17.26 ± 9.8%. The study was limited to single-dose efficacy testing and did not evaluate repeated dosing or systemic toxicity. Measurements using gold-loaded particles indicated organ exposure but did not establish the distribution or long-term clearance of intact MOF nanoparticles.

Hu et al. [214] developed an acid-resistant polyacrylamide copolymer hydrogel bearing adenine-rich and cytosine-rich DNA sequences with pH-responsive crosslinks. Parallel A-motif duplexes crosslinked the network at pH 1.2–3.0, whereas i-motif quadruplexes provided crosslinking at pH 4.0–6.0; both structures dissociated at pH 7.2. During sequential exposure to artificial gastric, duodenal, and intestinal fluids, fluorescence tracing indicated that insulin release remained at approximately 3% and 4% under gastric and duodenal conditions, respectively, followed by nearly complete release within 60 min in artificial intestinal fluid. An enzyme-linked immunosorbent assay recovered approximately 80% of the insulin in artificial intestinal fluid. In streptozotocin-induced diabetic rats, the orally administered formulation, which also contained the D-R8 cell-penetrating peptide and a trypsin inhibitor, lowered blood glucose for more than 12 h and achieved a relative insulin bioavailability of 5.3%. The observed effect therefore reflects the complete formulation and cannot be attributed to the hydrogel alone. Selected biochemical measurements and histological examination after 14 days did not identify abnormalities in the measured endpoints.

To combine gastric protection with intestinal absorption and hepatic insulin delivery, Hunt et al. [215] conjugated insulin to 3-mercaptopropionic acid-capped silver sulfide quantum dots (Ag_2_S QDs) and coated the conjugate with a chitosan/glucose copolymer, producing QD-INS-CS/GS. The formulation showed poor solubility under acidic conditions, whereas cellulase and β-glucosidase accelerated insulin release in vitro. In ex vivo human duodenal tissue, the tissue accumulated approximately 40-fold more ^14^C-associated signal from QD-INS-CS/GS than from free ^14^C-insulin after 2 h. After oral administration, radiotracer-associated signal was detected in the blood and liver of rodents, and oral QD-INS-CS/GS produced dose-dependent glucose lowering in mice. Its apparent oral bioavailability estimated from blood radioactivity was approximately 4%. Two-week oral treatment lowered blood glucose in non-obese diabetic mice and streptozotocin-induced diabetic rats. Hypoglycemia was not observed at the therapeutic regimens emphasized in the study, including an acute oral dose of 300 IU/kg in mice, although adverse hypoglycemia occurred at 500 IU/kg during dose-escalation testing, and six-week treatment in diabetic rats did not produce the weight increase observed in the injected-insulin comparator group. In non-diabetic baboons, single oral doses lowered blood glucose in a dose-dependent manner without recorded hypoglycemia or abnormal biochemical or hematological findings. Nevertheless, the human evidence was limited to ex vivo uptake, and the nonhuman primate experiment involved healthy animals and single doses. The radiotracer measurements did not distinguish intact insulin from labeled degradation products and did not track intact Ag_2_S QDs. The systemic disposition, clearance, and long-term safety of the complete formulation therefore remain unresolved.

A different GI-mediated strategy is to inhibit carbohydrate digestion within the intestinal lumen. Biomaterials can be used to deliver inhibitors of α-amylase, a key enzyme in carbohydrate digestion, for glycemic management. Guo et al. [216] generated α-amylase-specific egg-yolk immunoglobulin Y (IgY) with inhibitory activity against the enzyme. The IgY was covalently coupled to polyethylene glycol-coated Fe_3_O_4_ nanoparticles, which were embedded in pH-responsive chitosan-alginate hydrogel particles. The hydrogel limited IgY release in simulated gastric fluid and released most of the IgY-conjugated nanocomposite after transfer to simulated intestinal fluid. After oral gavage in diabetic rats, an external magnet generating 3,000 G was placed over the intestinal region to prolong particle retention. A single administration reduced postprandial glucose excursions during starch challenges through 36 h, but the effect was no longer evident at 48 h. Micro-computed tomography showed gastrointestinal retention of the formulation, and gastrointestinal-associated signals were no longer detected at 48 h. However, the study did not report serum biochemical or histological toxicity assessments and did not quantify tissue biodistribution, systemic exposure, iron burden, or longer-term material clearance.

Qi et al. [217] combined local digestive-enzyme inhibition with microbiota-related intervention. They developed an inulin and hyaluronic acid double-network hydrogel that co-delivered *Bifidobacterium longum (B. longum)* and nanoparticles composed of lactoferrin, zinc, and epigallocatechin gallate (EGCG). Protonation of hyaluronic acid reduced the hydrogel pore size under gastric conditions and protected the bacteria during simulated gastrointestinal exposure. In sequential in vitro testing, the hydrogel remained stable in simulated gastric and intestinal fluids but degraded in *B. longum* fermentation broth. The nanoparticle component retained free-radical-scavenging and α-glucosidase-inhibitory activities, while lactoferrin increased uptake by Caco-2 cells and adhesion to excised mouse intestinal mucosa. Oral administration before a sucrose challenge delayed the postprandial glucose increase, and fluorescence imaging showed greater gastrointestinal retention of the hydrogel formulation than of free nanoparticles at 6 h. In male mice with HFD- and streptozotocin-induced type 2 diabetes, daily oral treatment for 6 weeks lowered fasting glucose, glycated hemoglobin, and glycated serum protein and improved glucose tolerance and insulin sensitivity. These metabolic effects were accompanied by changes in intestinal barrier markers, colonic short-chain fatty acids, and microbial composition.

The material systems discussed in this subsection address three related objectives: local inhibition of carbohydrate digestion, modulation of the intestinal microbiota and mucosal environment, and intestinal delivery of peptide or protein therapeutics intended for systemic action. Individual formulations may combine more than one of these functions. However, consistent therapeutic effects require the delivery system to reach the intended intestinal region and release its cargo as designed across individuals. For microbiota-responsive systems, interindividual differences in microbial composition and metabolic activity may further affect drug release and treatment outcomes. All of the studies discussed here remain preclinical. Reproducibility across species, repeated-dose safety, carrier fate, and the clinical feasibility of retention using externally applied magnetic fields require further study. Representative preclinical in vivo studies across the disease areas are summarized in Table 2, with emphasis on administration route, model scope, mechanism, and selected outcomes.

**Table 2:** Representative preclinical in vivo studies of advanced biomaterials for gastrointestinal and gut-associated metabolic applications

Disease or indication	Material/intervention and administration route	Models and evidence scope	Main delivery or therapeutic mechanism	Selected in vivo findings	References
*Helicobacter pylori* infection	Verteporfin-loaded PLGA nanoparticles with a lecithin bilayer; oral administration followed by localized transabdominal ultrasound over the stomach	Female mice with gastric infection; efficacy and 48-h tolerability assessment	VacA adsorption and neutralization; ultrasound-triggered singlet-oxygen generation	Approximately 50-fold lower gastric bacterial burden than vehicle; efficacy comparable to triple therapy under the study conditions, with fewer short-term changes in fecal microbiota	[89]
Gastric ulcer	Sequentially pH-responsive PNMH polymer adhesive; local gastric application in rats and endoscopic delivery in pigs	Acetic acid-induced rat gastric ulcers; porcine gastric endoscopic submucosal dissection wounds	Acid-triggered gelation followed by molecular evolution that increased wet-tissue adhesion and limited pepsin transport to the wound	Complete healing of rat ulcers was reported by day 14; mean porcine wound-area closure at day 10 was 92.1%, versus 79.2% with saline and 73.4% with sucralfate	[13]
Gastric ulcer	Polyethyleneimine/poly(acrylic acid)/lactose-modified chitosan (PPCL) bioadhesive powder; direct application to surgically exposed rat ulcers	Acetic acid-induced rat gastric ulcers; rodent efficacy study; endoscopic delivery not tested	Rapid gelation, prolonged adhesion, acid and enzyme shielding, low swelling, and reactive oxygen species scavenging	Reduced oxidative stress and inflammation and promoted re-epithelialization, angiogenesis, and mucosal repair; the ulcer index was lower than that with aluminum phosphate gel at day 14	[96]
Gastric perforation	Acid-resistant brush-like ATGels bioadhesive; direct local sealing	Rat full-thickness gastric-perforation model; separate rat subcutaneous degradation study	Acid-resistant wet-tissue adhesion, defect sealing, and support of a pro-regenerative microenvironment	Improved histological repair compared with suturing over 28 days; in the separate subcutaneous study, less than 10% of the initial material mass remained at day 56	[107]
Gastric perforation	Vonoprazan- or acidic fibroblast growth factor-loaded mushroom-cap-inspired hydrogel occlusion device; surgical placement in rabbits and transesophageal endoscopic deployment in minipigs	Rabbit efficacy studies; minipig procedural feasibility, with one animal in each treatment group	Mechanical occlusion with acid suppression or repair support provided by separate drug-loaded formulations	Endoscopic placement achieved continued occlusion at 96 h in a minipig; the vonoprazan-loaded formulation maintained gastric pH above 4 for approximately 58 h, and the acidic fibroblast growth factor-loaded formulation improved healing markers in rabbits	[120]
Gastric perforation repair and prevention of adhesions	PACG/COS Janus hydrogel patch; direct placement over the defect	Rabbit gastric-perforation model; efficacy study	Tissue-facing wet adhesion combined with an outward-facing anti-adhesive surface	The patch adhered after 30 s of pressure and remained attached without sutures; no gross adhesion to adjacent tissue was observed at day 14	[123]
Inflammatory bowel disease	Infliximab-loaded, dually crosslinked Alg-AEMA microcapsules; oral administration	Dextran sulfate sodium-induced mouse colitis; efficacy study	Dual crosslinking to retain and release infliximab under the tested gastrointestinal conditions, followed by oral intestinal antibody delivery	Improved disease activity, colon length, histology, barrier markers, and inflammatory measures; several outcomes were broadly comparable with intravenous infliximab at the same dose. Treatment-associated changes in microbial richness were also observed	[131]
Inflammatory bowel disease	TMNR thermosensitive hydrogel containing Ga^3^⁺-chelated melanin nanoparticles; rectal enema	Dextran sulfate sodium-induced mouse colitis; efficacy study	Inflamed-colon retention, reactive oxygen species scavenging, Ga^3^⁺-associated interference with bacterial iron use, and physical barrier function	Improved disease activity, inflammatory cytokines, mucus-layer integrity, and goblet-cell numbers	[146]
Inflammatory bowel disease	Blood-responsive engineered *Escherichia coli* therapeutic living glue; enteric-coated oral formulation or rectal administration	Dextran sulfate sodium-induced and interleukin-10-knockout mouse colitis models; single-dose and repeated-dose studies	Bleeding-triggered bacterial production of the CP43K adhesive protein and trefoil factor 3	Improved weight recovery, reduced colon shortening and bleeding, and improved inflammatory and mucosal-barrier outcomes	[148]
Colorectal cancer	Oral HTPBD nanocomplexes carrying CRISPR-Cas9 plasmids	Orthotopic and chemoresistant orthotopic colorectal cancer models; spontaneous intestinal tumors in *Apc*^Min/+^ mice	Mucus penetration and transporter-mediated epithelial transcytosis; TRAP1 disruption enhanced 5-fluorouracil-induced tumor-cell death and antitumor immune responses	In an orthotopic model, HTPBD targeting TRAP1 plus 5-fluorouracil produced a reported 93.3% tumor-inhibition rate, versus 35.8% with 5-fluorouracil alone; in a chemoresistant model, addition of anti-PD-1 extended median survival to 44 days; intestinal tumor burden was reduced in *Apc*^Min/+^ mice	[161]
Colorectal cancer	Mitoxantrone-loaded ZIF-8 nanoparticles; intratumoral administration	Subcutaneous syngeneic CT26 tumors in BALB/c mice; local efficacy and immune profiling	Mitoxantrone chemotherapy combined with Zn^2^⁺-associated pyroptosis and amplified immunogenic cell death	Greater tumor-growth inhibition than free mitoxantrone; complete remission in 2 of 6 mice and prolonged survival	[194]
Nonalcoholic fatty liver disease	pH- and microbiota-responsive nitidine-chloride-loaded chitosan/pectin nanoparticles; oral gavage for 4 weeks	Male C57BL/6 mice with high-fat diet-induced nonalcoholic fatty liver disease; efficacy study	pH- and microbiota-responsive release under simulated colonic conditions; treatment-associated changes in gut microbiota	Reduced weight gain and serum aminotransferase and lipid levels; attenuated hepatic and intestinal inflammatory and histological changes compared with untreated high-fat diet controls	[210]
Diabetes	Enteric capsule containing zwitterionic-hydrogel-coated NH_2_-MIL-101(Fe) loaded with exendin-4; oral administration	Diabetic rats; single-dose pharmacokinetic and pharmacodynamic study	Enteric protection and intestinal release; zwitterionic coating designed to reduce mucus binding and enhance epithelial uptake	Maintained elevated plasma exendin-4 for more than 8 h, increased endogenous insulin secretion, and lowered blood glucose; relative pharmacological availability was 17.26 ± 9.8%	[47]
Diabetes	Ag_2_S quantum dot–insulin formulation coated with a chitosan/glucose copolymer (QD-INS–CS/GS); oral administration	Diabetic mice and rats; single-dose studies in non-diabetic baboons; supporting human duodenal tissue evaluated ex vivo	Protection under acidic conditions, glucosidase-responsive insulin release, intestinal absorption, and hepatic delivery of insulin	Produced dose-dependent glucose lowering in diabetic mice and rats. Hypoglycemia was not observed at the principal therapeutic doses. Six-week treatment did not produce the weight gain observed with injected insulin in diabetic rats, and single oral doses lowered blood glucose in non-diabetic baboons	[215]

All entries are preclinical. Human duodenal tissue in the Hunt study was evaluated ex vivo, and the baboon study used non-diabetic animals. Large-animal procedural feasibility does not establish clinical efficacy. Results from different formulations or disease models are stated separately

*Abbreviations*: *Alg-AEMA* aminoethyl methacrylate-modified alginate, *ATGels* an author-defined designation for an acid-tolerant brush-like hydrogel bioadhesive, *COS* chitooligosaccharide, *CP43K* an engineered adhesive protein, *CRISPR-Cas9* clustered regularly interspaced short palindromic repeats and CRISPR-associated protein 9, *GI* gastrointestinal, *HTPBD* hyaluronic acid- and trimethylamine N-oxide-modified poly(β-amino ester)-based DNA nanocomplex, *PACG* poly(N-acryloyl-2-glycine), *PD-1* programmed cell death protein 1, *PLGA* poly(lactic-co-glycolic acid), *PNMH* the copolymer obtained from β-carboxylic-amide-functionalized N-acryloyl phenylalanine and hydroxyethyl acrylamide, *PPCL* polyethylenimine/poly(acrylic acid)/lactose-modified chitosan, *QD* quantum dot, *ROS* reactive oxygen species, *TMNR* targeted metabolic nanoregulator, *TRAP1* tumor necrosis factor receptor-associated protein 1, *VacA* vacuolating cytotoxin A, *ZIF-8* zeolitic imidazolate framework-8

## Clinical translation: challenges and opportunities

**Fig. 3:**
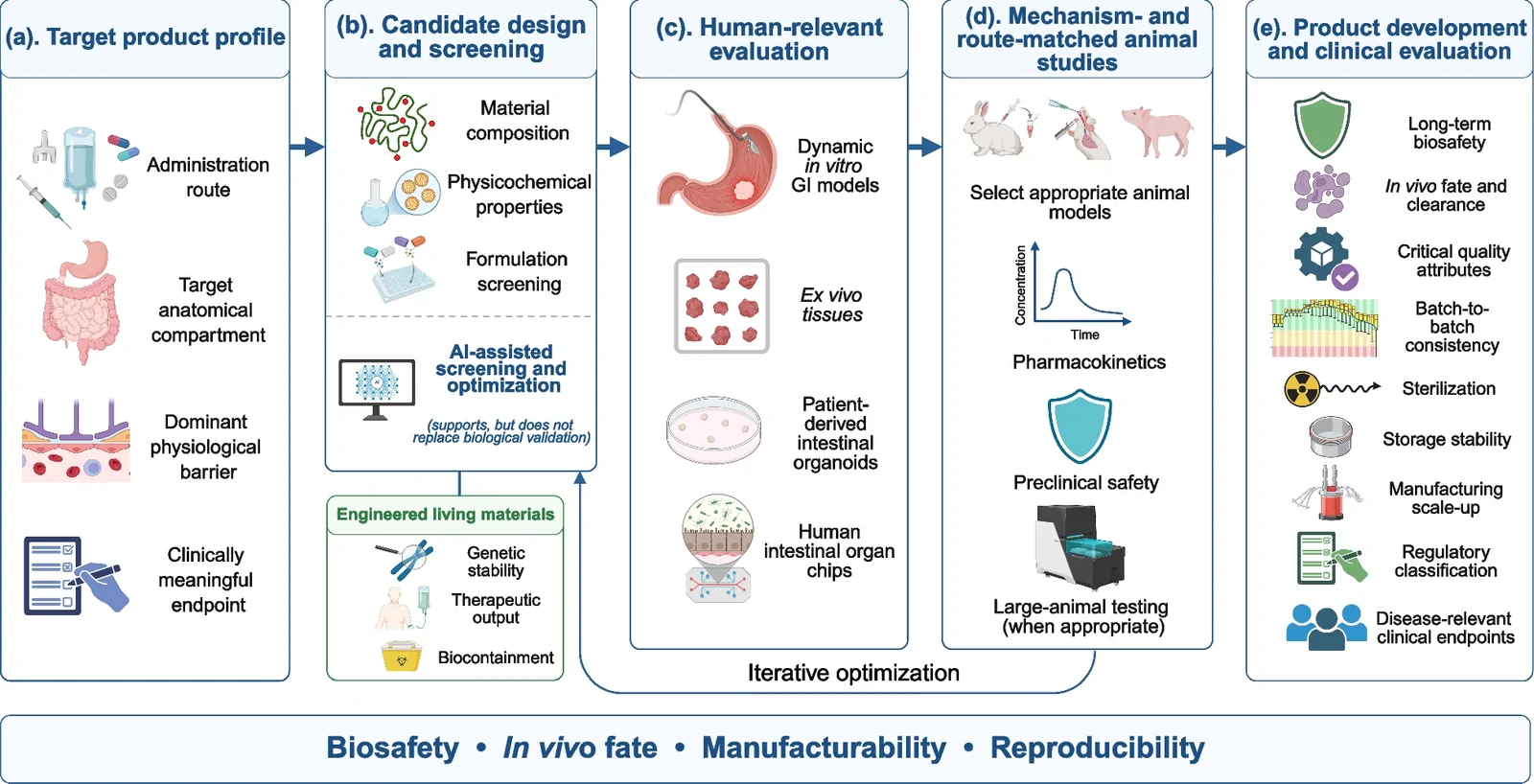
Development and clinical translation of gastrointestinal biomaterials. **a** The target product profile specifies the intended administration route, anatomical target, dominant physiological barrier, and clinically meaningful endpoint. **b** Candidate design and screening consider material composition, physicochemical properties, and formulation performance. **c** Human-relevant evaluation integrates dynamic in vitro gastrointestinal models, ex vivo tissues, patient-derived intestinal organoids, and human intestinal organ-on-chip systems. **d** Appropriately selected animal models are used for pharmacokinetic and preclinical safety assessment. **e** Product development and clinical evaluation address the principal safety, manufacturing, regulatory, and clinical requirements for translation. The feedback loop denotes iterative optimization, while biosafety, in vivo fate, manufacturability, and reproducibility are considered throughout development. Abbreviations: AI, artificial intelligence; GI, gastrointestinal. Created in BioRender

Despite substantial preclinical progress, the clinical translation of GI-targeted biomaterials remains slow. Much of the evidence reviewed here comes from in vitro studies and rodent models, whereas evidence from large-animal studies and human clinical trials remains comparatively limited [8, 40]. Preclinical efficacy alone does not establish product viability. A candidate must be compatible with the intended administration route, be reproducibly manufactured at scale, show an acceptable safety profile for the intended route and dosing schedule, and provide clinically meaningful benefit relative to appropriate clinical comparators [40]. Translation planning should accompany material optimization. Key considerations include the human relevance of preclinical evidence, biosafety and manufacturability, and the regulatory and clinical evidence needed for development. These considerations are integrated into the staged and iterative framework for the development and clinical translation of GI biomaterials summarized in Fig. 3.

### Preclinical-clinical gap and human-relevant evaluation

The translation of GI biomaterials from preclinical proof-of-concept studies to clinical use is complicated by differences between commonly used animal models and humans in GI motility, luminal pH, digestive and metabolic activity, and microbiome composition [20, 218, 219]. The suitability of a model therefore depends on the administration route, target GI compartment, and material function being evaluated [8, 40]. GI motility differs markedly among species. Reported gastric emptying and intestinal transit times vary with species, GI region, feeding status, dosage-form properties, and measurement methods [218, 220]. These differences affect drug residence and carrier performance. Luminal pH also differs among species and varies with feeding status and measurement conditions. Measurements in humans and commonly used animal models, including mice, rats, rabbits, guinea pigs, and pigs, have demonstrated species-specific gastric and intestinal pH profiles [21, 221, 222]. These regional differences can alter polymer dissolution, carrier disassembly, and site-specific release. The composition and enzymatic activity of luminal fluids also vary across species and physiological conditions. Direct comparisons have shown substrate-dependent differences in prodrug degradation between human and canine intestinal fluids, while human duodenal fluids show substantial interindividual and feeding-related variation in lipase, phospholipase, and esterase activities [223, 224]. Intestinal and hepatic cytochrome P450 (CYP) isoforms and activities differ between humans and laboratory animals [225, 226]. These differences can affect the luminal degradation of susceptible drug molecules and, after absorption, first-pass metabolism and systemic exposure.

Gut microbial communities differ across host species. Comparative analyses of mice, rats, nonhuman primates, and humans show that their gut microbiota share some higher-level taxa but differ markedly in the relative abundance and composition of specific genera and species, as well as in microbial gene content and fecal metabolite profiles [227, 228]. Studies of microbiome-targeted interventions must account for these interspecies differences. Germ-free mice and mice colonized with human microbiota enable controlled mechanistic experiments. However, human microbiota-associated mice do not fully reproduce the donor microbial community or the interactions between the human host and microbiota [229]. Gut microbiota composition also varies among vendors, production sites, and animal facilities, which can affect experimental phenotypes and reproducibility across preclinical studies [227, 230, 231].

Large-animal models are particularly informative when the translational question depends on anatomical scale, clinical deployment, intraluminal transit or retention, or mechanical interaction with moving GI tissue [40, 219, 232, 233]. These models can test whether a formulation or device can be delivered using clinically relevant equipment, remain at the intended site, and function under conditions that are difficult to reproduce in rodents. Pigs have GI dimensions and mucosal anatomy that support endoscopic and device-based testing, although their gastric pH, gastric emptying, and intestinal transit do not consistently reproduce human values [218, 222, 233]. For oral absorption studies, pigs, dogs, and nonhuman primates have different physiological advantages and limitations. Therefore, model selection should reflect the dosage form and absorption mechanism rather than a general assumption of similarity to humans [218, 233]. Selected GI measurements in fasted cynomolgus monkeys resemble published human values and support their use for specific oral dosage-form studies [234]. Berg et al. [235] evaluated an intestinal device for focal intestinal peptide delivery in pigs, and Ying et al. [236] tested an endoscopically delivered electroadhesive hydrogel for prolonged contact with porcine GI mucosa. Model choice should be matched to the administration route, product characteristics and translational question [40]. No animal model, however, fully reproduces human GI physiology or disease heterogeneity [20, 218, 237].

Ex vivo models provide additional tools for assessing biomaterial permeability and tissue interactions. Ussing chambers can be used to measure transport across the intestinal epithelium and assess epithelial barrier function, whereas precision-cut intestinal slices retain multiple intestinal cell types and can support studies of drug transport, metabolism, and local toxicity [238, 239]. A high-throughput porcine ex vivo intestinal platform has been used to compare albumin variants and nanoparticle formulations targeting the neonatal Fc receptor (FcRn) for transmucosal transport [240], and Caco-2 monolayers overlaid with ex vivo porcine intestinal mucus can support permeation and compatibility studies [241]. Patient-derived organoids can retain donor-specific epithelial features, while co-culture and organ-on-chip models can further incorporate selected immune cells, microorganisms, vascular components, fluid flow, and mechanical stimulation [242, 243]. These systems reproduce selected components of intestinal biology but do not independently capture whole-body pharmacokinetics or the full complexity of human disease.

Machine-learning methods can assist candidate screening and formulation design. They have been used to predict formulation release, screen ionizable lipid libraries, guide the design of adhesive hydrogel formulations, and analyze drug–transporter interactions in intestinal tissue [244–247]. However, their performance with new materials and experimental settings still needs to be confirmed experimentally [248]. Organ-on-chip models serve a different purpose. Intestinal and multi-organ chip systems have reproduced selected epithelial, mucus, and microbiome features and have supported quantitative prediction of human pharmacokinetic responses [249–252]. Before an organ-on-chip model is used, the question it is intended to answer should be defined. Its performance and reproducibility should then be compared with appropriate reference methods and clinically relevant measurements [253].

No single model can provide all the evidence required for clinical translation. Model selection should be based on the question being addressed [40, 219, 242]. In vitro and ex vivo systems can clarify mechanisms and local tissue interactions. Organoids and organ-on-chip models can reproduce selected features of human tissue [242]. Animal models can provide information on whole-body exposure and performance under the intended route of administration [219]. When a material is delivered with a device or through a specific clinical procedure, testing should confirm that it can be delivered and retained at the intended site [40].

### Long-term biosafety, manufacturing, and quality control

Long-term safety cannot be inferred from short-term cytocompatibility tests or short-term histological findings alone [40, 254]. Safety studies should use test articles representative of the intended final formulation and should reflect the intended administration route and dosing schedule [40, 254]. Relevant factors include material composition, dose, dosing frequency, treatment duration, process-related impurities, and the absorption, distribution, persistence, transformation, and elimination of material-associated components [62, 255]. For multicomponent products, the carrier, payload, surface coating, and major degradation or transformation products should be characterized separately where feasible. When systemic exposure or persistence is plausible, repeated-dose studies should determine whether material-associated components accumulate locally or systemically and whether their concentrations or tissue burdens decline after dosing stops [40, 255].

The mechanisms and rates of degradation in polymer carriers and hydrogels depend on material chemistry and structure [256, 257]. In degradable systems containing susceptible bonds, hydrolysis or enzyme-mediated reactions can cleave polymer chains or hydrogel crosslinks. In water-insoluble matrices, this bond cleavage can lead predominantly to surface erosion or bulk erosion, depending on the relative rates of fluid penetration and bond cleavage. Polymer composition, molecular weight, crystallinity, water uptake, crosslink density, and matrix geometry can affect degradation, whereas drug release also depends on payload size and interactions between the payload and polymer network [256, 257]. Loss of the visible matrix or a decrease in mass does not identify the chemical products formed and, by itself, does not demonstrate that those products have been eliminated from the body [40, 255, 258]. Major degradation products should therefore be identified and assessed for local effects and elimination; biodistribution and clearance studies are warranted when systemic absorption is plausible [40, 255].

For inorganic nanoparticles and metal–organic framework (MOF) composites, loss of the original particle or framework structure does not necessarily mean that all components have been eliminated. Depending on their composition and biological environment, inorganic particles may dissolve and release ions, undergo oxidation, reduction, or complexation reactions, form new metal-containing species, or persist in particulate form [62, 259]. The inorganic core, engineered surface coating, and adsorbed biomolecular corona may change or separate over time and can therefore have different distributions and elimination pathways [62]. For MOFs, stability and disassembly depend on metal–ligand chemistry and framework structure, as well as particle size, surface properties, and the pH, ions, and proteins in the surrounding medium [64, 260]. Zhang et al. [65] observed greater structural collapse of Pd(H)@ZIF-8 after 12 h in simulated gastric fluid than in phosphate-buffered saline and separately detected hydrogen release using a methylene blue assay. The authors proposed that acid-induced disassembly of ZIF-8 released Zn^2^⁺, which contributed to the observed antibacterial activity. However, the study did not directly characterize the chemical species generated during framework disassembly or determine the absorption, organ distribution, and excretion of the palladium-containing material. The observed structural collapse therefore supports acid-responsive disassembly of the framework, but not complete degradation or clearance of the composite.

Biologically derived and living products raise different safety and quality concerns. For decellularized extracellular matrix (ECM) products, evaluation should consider tissue source, residual cellular components and DNA, process residues, processing-induced changes to matrix structure and function, potential pathogens, and host immune responses. Residual DNA is commonly used as one indicator of decellularization efficiency, but a low DNA level alone does not demonstrate safety or low immunogenicity because other cellular remnants, process residues, matrix alterations, and implantation conditions also influence the host response [69, 261]. For cell membrane-coated nanoparticles, quality control should verify the identity and purity of the source cells and isolated membranes and assess the membrane-to-core ratio, coating completeness and orientation, preservation and function of relevant membrane proteins, batch consistency, and microbial contamination and endotoxin levels, where applicable [66, 262]. Engineered live bacterial products require confirmation of strain identity, genetic stability, viable-cell count, and potency linked to the intended therapeutic function [263–265]. Studies should also determine how long the bacteria persist, how they are eliminated, whether they spread beyond the intended site, and, where relevant, whether antibiotic-resistance genes or engineered DNA could be transferred to other microorganisms. Biocontainment efficacy should be evaluated by testing for survival or proliferation of engineered bacteria at non-target sites or in the external environment [263, 264].

Material-specific risks should be evaluated in the context of the administration route and treatment duration [40]. For nonabsorbed oral and intraluminal products, relevant endpoints may include local mucosal tolerance and effects on gastrointestinal transit. Gastroretentive devices should also be assessed for unintended retention and gastrointestinal obstruction [8, 37]. When a product is expected to interact with resident microorganisms, studies should evaluate intended and unintended changes in microbiome composition and function. Materials applied locally or endoscopically may require assessment of tissue injury, unintended adhesion, and effects on luminal patency or motility, depending on their size and residence time [8, 37]. For temporary materials, removal or degradation after their intended function is complete should also be evaluated [40, 103]. For systemically administered nanoparticles, safety studies should characterize circulation and biodistribution, interactions with blood and immune components, and major-organ exposure under the intended dosing schedule [33, 40, 255, 266]. Safety endpoints should therefore be selected according to material composition, administration route, treatment duration, and expected local or systemic exposure rather than applied as a uniform checklist [40].

The relevance of nonclinical safety results to clinical development depends on whether the tested material adequately represents the formulation and manufacturing process intended for patient use [40, 267]. For nanoparticle formulations, changes in mixing, shear, temperature, solvent handling, purification, and drying during scale-up can alter particle-size distribution, drug loading, release behavior, impurity levels, and stability [40, 219, 268, 269]. Biologically sourced products introduce additional variation through the source material, isolation or purification process, and preservation method [66, 69, 270]. Engineered live bacterial products must also retain their identity, viability, and potency during manufacture [263]. Formulation and manufacturing-process development should proceed in parallel, and comparability across representative batches should be demonstrated using relevant quality attributes as development advances [40, 267, 271].

Quality by design (QbD) provides a structured approach for linking the intended dosage form and clinical function to measurable product attributes and process controls [271, 272]. Critical quality attributes (CQAs) should be defined for each product, followed by identification of the material attributes and process parameters that influence them [40, 271, 273]. For hydrogels and tissue adhesives, relevant CQAs may include composition, gelation or deployment time, swelling, mechanical properties, wet-tissue adhesion, drug release when a payload is incorporated, and degradation after use [103, 274, 275]. For particle formulations, relevant CQAs may include composition, particle-size distribution, morphology, surface properties and, where applicable, coating properties, drug loading, the proportion of unencapsulated drug, in vitro release, process-related impurities, aggregation, and physical and chemical stability [40, 254, 267, 276]. Microbiological quality should also be controlled, with sterility and bacterial endotoxin requirements determined by the dosage form, administration route, and product type [267, 277]. ECM-derived, cell membrane-coated, and engineered bacterial products require additional controls related to biological source, identity, purity, potency, batch consistency, viability, or biocontainment, as applicable [66, 69, 263]. These examples are product-specific and should not be treated as a universal testing panel [267, 271].

Sterilization and storage should be considered early in product development [40]. For products required to be sterile, the sterilization process should be selected according to formulation composition and physical form, the sensitivity of the material and therapeutic cargo to heat, radiation, or reactive chemicals, and compatibility with the proposed container-closure system; no method is suitable for every formulation. Terminal sterilization by gamma irradiation, electron-beam irradiation, or ethylene oxide treatment can be used for some products, but compatibility with the final material system must be demonstrated [274, 278]. For filterable solutions that cannot tolerate terminal treatment, sterilizing filtration followed by aseptic processing may be used [278, 279]. In hydrogels, some sterilization methods can cause polymer-chain scission or additional crosslinking and can alter swelling, mechanical properties, biocompatibility, drug stability, and release behavior [274, 278]. The selected process should therefore be validated using the final formulation and proposed container-closure system [278, 279].

Stability studies should evaluate the intended dosage form in its proposed container-closure system and should include in-use testing after reconstitution or dilution when applicable [280]. Compatibility with the intended administration device should also be assessed when it may affect dose delivery or product quality. Product-specific stability tests should monitor attributes likely to change during storage or use, including chemical integrity and potency, degradation products, particle aggregation or phase separation, mechanical or deployment performance, release behavior, and microbiological quality, as applicable [40, 280]. Clinical readiness ultimately depends on whether the final product can be manufactured reproducibly and retain its specified quality, performance, and stability throughout storage and use [40].

### Regulatory classification and clinical translation landscape

Depending on their composition, intended use, and jurisdiction, drug-loaded hydrogels, device-assisted capsules, living therapeutic matrices, and image-guided magnetically controlled delivery systems may fall under different regulatory categories, including drugs, medical devices, biological products, and combination products. In the United States, this classification affects the required nonclinical studies, manufacturing oversight, and premarket pathway. For a combination product, the primary mode of action is the single mode of action expected to make the greatest contribution to the product’s overall intended therapeutic effects. If the primary mode of action or product classification is unclear, early consultation with the U.S. Food and Drug Administration (FDA) is advisable [281]. Limiting a design to the components needed for its intended clinical function can reduce characterization demands and ease manufacturing scale-up and regulatory assessment [40, 282, 283].

Clinical endpoints should correspond to the expected benefit. Studies of ulcer coatings should distinguish product-performance endpoints, such as residence at the lesion, from clinical outcomes appropriate to the intended function. Repair-oriented products should assess ulcer healing, whereas hemostatic products should be evaluated for initial bleeding control, recurrent bleeding, and the need for repeat intervention [284]. Perforation sealants should be assessed for successful closure, persistent or recurrent leakage, procedure-related adverse events, and the need for surgical rescue [99]. IBD delivery systems may be evaluated using symptom-based and endoscopic outcomes, with histological outcomes as adjuncts [285]. Corticosteroid use and systemic safety should also be monitored to provide a more comprehensive assessment of the clinical benefits and risks of the treatment. Drug exposure at the target site can be used to assess pharmacokinetics and delivery performance but should not be regarded as a clinical outcome. Material-assisted tumor treatments should be evaluated using disease- and setting-appropriate outcomes, such as tumor response, recurrence, survival, and treatment-related toxicity, with comparison against standard care when appropriate. Exploratory biomarker changes alone should not be interpreted as established clinical benefit [286]. The literature reviewed here shows a clear gap between comparatively established GI formulations or materials and complex responsive platforms, many of which remain at the preclinical stage [8, 40].

Site-directed oral delivery is already used clinically in budesonide formulations. ENTOCORT EC contains enteric-coated granules that dissolve at pH values above 5.5 and is indicated in the United States for the treatment of mild-to-moderate active Crohn’s disease involving the ileum and/or ascending colon in patients aged 8 years and older [287]. UCERIS uses a coating that dissolves at pH values of 7 or above, followed by extended release from a polymer matrix, and is indicated in the United States for the induction of remission in active mild-to-moderate ulcerative colitis [288, 289]. The FDA-cleared PuraStat self-assembling peptide gel [290] and EndoClot polysaccharide hemostatic system [291] are examples of endoscopically delivered materials cleared for specified nonvariceal GI bleeding indications. In a prospective multicentre cohort, initial haemostasis was achieved in 74 of 79 patients who received PuraStat as primary therapy [292].

Clinical outcomes have also been mixed for oral macromolecule formulations. A 28-day randomized trial of the oral insulin candidate ORMD-0801 reported improvements in some short-term glycemic measures and a safety profile similar to that of placebo [293]. However, according to sponsor-reported top-line results, the phase 3 ORA-D-013-1 trial (NCT04606576) [294] did not meet its primary glycated hemoglobin (HbA1c) endpoint or its secondary fasting plasma glucose endpoint at week 26 [295]. The parallel ORA-D-013-2 trial (NCT04754334) was subsequently terminated on the basis of those results [296]. AMT-101, a GI-selective oral interleukin-10 (IL-10) fusion protein, reached phase 2 a evaluation. Sponsor-reported results indicated that the LOMBARD trial (NCT04583358) [297] did not outperform placebo and that the MARKET trial (NCT05372939) [298] showed no overall added clinical benefit when AMT-101 was combined with adalimumab [299]. These examples show that early pharmacodynamic activity and short-term tolerability do not necessarily translate into clinical efficacy.

Overall, the evidence summarized in Tables 2 and 3 indicates that most advanced GI biomaterial systems discussed in this review remain preclinical. The limited cleared devices and clinical-stage GI delivery systems do not yet establish broad clinical maturity. Clinical progression should depend on product quality, safety, manufacturability, and clinically meaningful benefit rather than multifunctionality alone [40, 283, 300]. Representative examples are summarized in Table 3.

**Table 3:** Representative cleared devices and clinical-stage gastrointestinal delivery systems

Disease or setting	Product or study	Delivery principle and route	Regulatory or trial status	Evidence and interpretation	References
Mild-to-moderate bleeding after endoscopic submucosal dissection or mucosal resection, and primary nonvariceal GI bleeding	PuraStat (synthetic self-assembling RADA16 peptide gel)	Endoscopically delivered peptide solution that rapidly forms a hydrogel matrix barrier on contact with physiological fluid	U.S. FDA 510(k)-cleared (K222481, 2023; predicate PuraStat-GI K210098, 2021)	Cleared for specified post-procedural and primary nonvariceal GI bleeding; not indicated for arterial Forrest Ia bleeding	[290]
Nonvariceal GI bleeding	EndoClot Polysaccharide Hemostatic System	Endoscopically delivered absorbable modified polymer particles derived from plant starch	U.S. FDA 510(k)-cleared (K190677, 2021)	Cleared for hemostasis of nonvariceal GI bleeding, excluding Forrest Ia bleeding	[291]
Type 2 diabetes with inadequate glycemic control	ORMD-0801 (ORA-D-013-1 and ORA-D-013-2)	Oral enteric-coated insulin capsule containing a protease inhibitor and an absorption enhancer	Phase 3; both registry records list the trials as Terminated (NCT04606576; NCT04754334)	ORA-D-013-1 enrolled 710 participants and, according to the sponsor, did not meet the primary HbA1c endpoint or secondary fasting plasma glucose endpoint at week 26. ORA-D-013-2 enrolled 346 participants and was terminated following the ORA-D-013-1 readout	[294–296]
Moderate-to-severe ulcerative colitis	AMT-101 (LOMBARD)	Oral GI-selective recombinant human interleukin-10 fusion designed for active transport across the intestinal epithelium into local GI tissue	Phase 2; ClinicalTrials.gov status Unknown (NCT04583358; last updated 7 November 2022)	105 participants were randomized. Sponsor-reported week-12 clinical remission was 17.1% (12/70) with AMT-101 versus 20.0% (7/35) with placebo; the efficacy endpoint was not met. No results are posted in the registry	[297, 299]
Moderate-to-severe ulcerative colitis	AMT-101 plus adalimumab (MARKET)	Oral GI-selective interleukin-10 fusion plus subcutaneous anti-tumor-necrosis-factor therapy	Phase 2; Completed (NCT05372939)	51 participants were randomized. Sponsor-reported week-8 clinical remission was 31.8% (7/22) with AMT-101 plus adalimumab versus 33.3% (9/27) with adalimumab plus placebo; no added overall clinical benefit was shown. No results are posted in the registry	[298, 299]

Devices reviewed through 510(k) are described as FDA-cleared. Clinical trial registry status and enrollment were checked on 17 September 2026. Registry status is dynamic. Where no results were posted in ClinicalTrials.gov, outcome statements are explicitly identified as sponsor-reported and supported by an SEC filing or named sponsor report

*Abbreviations*: *FDA* U.S. Food and Drug Administration, *GI* gastrointestinal, *HbA1c* glycated hemoglobin

## Conclusion and future perspectives

Advanced biomedical materials have expanded the strategies available for GI drug delivery, tissue repair, microenvironment modulation, and interventions for gut-associated metabolic disorders. Across the disease-specific applications reviewed here, biomaterials mainly contribute through cargo protection, local retention or transport, site-selective release, tissue repair, and immune or microbiome modulation. Because the limiting factors differ by clinical indication, administration route, anatomical site, and intended endpoint, no single material system can address every GI barrier, and adding functions does not by itself increase translational value. Material design should begin by defining the clinical indication, the administration route, target compartment, dominant physiological barrier, and clinically relevant endpoint. Oral and intraluminal systems must balance cargo protection, regional release, mucosal residence, and clearance. Local or endoscopically delivered materials require reliable deployment, wet-tissue adhesion, and controlled degradation, whereas systemic carriers must remain stable in circulation, reach the lesion, and limit off-target exposure.

Translation remains limited by the predominance of short-term rodent studies, incomplete evidence on human relevance and long-term material fate, and limited assessment of repeated administration. Clinically meaningful comparators and endpoints are not always defined, while manufacturing and regulatory requirements are often considered late in development. Before material optimization begins, future programs should establish a target product profile that specifies the intended patient population, route, dose and schedule, comparator, and clinical endpoint [40]. Testing should then use route-relevant measures, such as stability and regional release for oral systems, deployment and retention for local materials, or exposure and biodistribution for systemic carriers. Candidates should advance only when they show reproducible performance against an appropriate comparator, acceptable safety under the intended dosing schedule, and feasible manufacture. Critical quality attributes, scale-up, sterilization, and storage stability should be assessed early so that unsuitable candidates can be discontinued before extensive development.

Alongside these improvements in study design and product development, emerging tools may support candidate selection and human-relevant evaluation. Artificial intelligence (AI) can assist candidate screening and formulation optimization, but predictions based on small or inconsistent datasets require experimental confirmation before they guide further development [248]. Organ-on-chip models serve a different role by allowing selected candidates to be evaluated under defined, human-relevant conditions. Their intended use should be specified in advance, and their reproducibility and predictive performance should be compared with established methods or relevant clinical measurements [253]. These tools may make candidate selection and evaluation more efficient, but they cannot replace whole-body pharmacokinetic studies, safety testing, or clinical evaluation.

Whereas AI and organ-on-chip models support material development and evaluation, engineered living materials represent a distinct therapeutic platform. They may enable local, programmable production of therapeutic molecules within the GI tract. However, they introduce risks that conventional carrier testing may not capture, including genetic instability, uncontrolled output, unintended persistence, fecal shedding, horizontal gene transfer, and environmental survival. Because auxotrophic containment can be breached during intestinal residence and viable auxotrophic bacteria may persist after excretion, future systems should combine independent containment mechanisms with inducible kill switches [301, 302]. Therapeutic output, genetic stability, persistence, shedding, and containment failure should have prespecified limits. Development should proceed only when these limits are met and control remains effective both during treatment and after excretion.

Despite these different technological directions, the same principle should guide their development: Each additional component should address a defined clinical limitation or requirement, and a complex system should be compared with a simpler design intended for the same clinical purpose. Candidates should be judged by reproducibility, manufacturability, performance in human-relevant models, safety, and clinically meaningful patient benefit. Complexity is justified only when it improves a relevant endpoint sufficiently to offset the added burdens of production, quality control, safety assessment, and regulation. For many GI indications, the most translatable biomaterial may therefore be the simplest system that reliably performs its intended task for the intended route and patient population. Future progress in biomaterial development will depend less on increasing material complexity. Instead, materials should be designed to meet specific clinical needs and offer clear advantages over simpler alternatives.

## Data Availability

Not applicable. All data analyzed in this review are from previously published studies, which are cited in the reference list. No new datasets were generated or analyzed during the preparation of this manuscript.
